# IK_Ca_ channels control breast cancer metabolism including AMPK-driven autophagy

**DOI:** 10.1038/s41419-022-05329-z

**Published:** 2022-10-27

**Authors:** Dominic Gross, Helmut Bischof, Selina Maier, Katharina Sporbeck, Andreas L. Birkenfeld, Roland Malli, Peter Ruth, Tassula Proikas-Cezanne, Robert Lukowski

**Affiliations:** 1 Department of Pharmacology, Toxicology and Clinical Pharmacology, Institute of Pharmacy, University of Tübingen, Tübingen, Germany; 2 Department of Molecular Biology, Interfaculty Institute of Cell Biology, University of Tübingen, Tübingen, Germany; 3 Institute of Diabetes Research and Metabolic Diseases (IDM), the Helmholtz Center, Munich, Germany; 4 Department of Internal Medicine IV, Division of Endocrinology, Diabetology and Nephrology, University Hospital of Tübingen, Tübingen, Germany; 5 Gottfried Schatz Research Center for Cell Signalling, Metabolism and Aging, Division of Molecular Biology and Biochemistry, Medical University of Graz, Graz, Austria; 6 BioTechMed-Graz, Graz, Austria

**Keywords:** Autophagy, Potassium channels, Breast cancer, Cellular imaging, Cancer metabolism

## Abstract

Ca^2+^-activated K^+^ channels of intermediate conductance (IK) are frequently overexpressed in breast cancer (BC) cells, while IK channel depletion reduces BC cell proliferation and tumorigenesis. This raises the question, of whether and mechanistically how IK activity interferes with the metabolic activity and energy consumption rates, which are fundamental for rapidly growing cells. Using BC cells obtained from MMTV-PyMT tumor-bearing mice, we show that both, glycolysis and mitochondrial ATP-production are reduced in cells derived from IK-deficient breast tumors. Loss of IK altered the sub-/cellular K^+^- and Ca^2+^- homeostasis and mitochondrial membrane potential, ultimately resulting in reduced ATP-production and metabolic activity. Consequently, we find that BC cells lacking IK upregulate AMP-activated protein kinase activity to induce autophagy compensating the glycolytic and mitochondrial energy shortage. Our results emphasize that IK by modulating cellular Ca^2+^- and K^+^-dynamics contributes to the remodeling of metabolic pathways in cancer. Thus, targeting IK channel might disturb the metabolic activity of BC cells and reduce malignancy.

## Introduction

Tumor cells display a profound increase in metabolic activity to compensate for their accelerated and unconstrained cell proliferation rates [1, 2]. To supply the increased energy demand, cancer cells exhibit unique metabolic peculiarities such as the *Warburg effect*, which refers to a high rate of glycolysis despite the presence of molecular oxygen (O_2_) [1, 3, 4]. This process gradually converts glucose to pyruvate to produce adenosine-5′-triphosphate (ATP). Interestingly, however, in cancer cells, pyruvate is reduced to lactic acid, which is then secreted, while non-malignant cells usually metabolize pyruvate via the tricarboxylic acid (TCA) cycle [1]. Besides glycolysis, catabolic processes like autophagy maintain energy supply by providing nutrients through the digestion of cell debris and proteins [5]. Depending on the ratio of adenosine-5′-monophosphate (AMP) and adenosine-5′-diphosphate (ADP) to ATP, AMP-activated protein kinase (AMPK), which consists of a trimeric complex containing catalytic subunit (α-subunit) and two regulatory subunits (β- and γ-subunits), controls the maintenance of energy homeostasis [6–8]. Binding of AMP or ADP to the γ-subunit results in a conformational change of AMPK, allowing kinases such as liver kinase B1 (LKB1) to phosphorylate AMPK at threonine 172 (Thr^172^) [7–9]. A calcium ion (Ca^2+^)-dependent regulation of AMPK activity, which is primarily induced via phosphorylation of the α-subunit of AMPK by Ca^2+^/calmodulin-dependent protein kinase kinase 2 (CaMKK2), was also demonstrated [7, 10]. The activation of AMPK by these mechanisms leads to diminished anabolic processes and initiation of catabolic processes such as autophagy to ensure cellular energy supply [11].

In addition to energy production, cellular energy homeostasis is also determined by energy consumption. Processes including cell proliferation, migration, vesicular transport, and maintenance of the intracellular ion homeostasis consume high levels of energy [1, 12–14]. Potassium ion (K^+^) channels have been shown to impact these effects and their expression is dysregulated in multiple cancer entities [15–17]. K^+^ channels can accelerate tumor development by affecting the plasma- and mitochondrial membrane potential (ΔΨ_mem_, ΔΨ_mito_), vesicle secretion, or even glycolytic activity [17–19]. Pharmacological blockade of discrete members of the K^+^ channel family has shown potent anti-cancer properties, suggesting that targeting these channels may improve cancer treatment in vivo [15, 17, 20, 21]. Among other K^+^ channels [15], the Ca^2+^-activated K^+^ channel (K_Ca_) subfamily members of the big (BK) and intermediate (IK *aka* IK_Ca_, KCNN4, K_Ca_3.1 or SK4) type have been proposed as therapeutic targets in breast cancer (BC) [22, 23]. BK may account for women’s risk of BC and the outcome of anti-hormonal therapy [23], while the oncogenic role of endogenous IK channels was recently validated in a MMTV-PyMT-induced BC mouse model [24]. Absence of IK increased BC cell and tumor sensitivity to radiotherapy [25] and resulted in a reduced growth factor-dependent Ca^2+^ entry, cell cycle arrest and a decreased rate of proliferation in MMTV-PyMT tumor-derived BC cells [22, 24, 26]. Besides its localization in the plasma membrane, IK was also identified within the inner mitochondrial membrane (IMM) of various cancer cell lines, but the role of mitochondrial IK (mitoIK) channels is less well defined [19].

Based on these previous findings, we examined, whether and by which mechanisms IK depletion modulates the metabolic homeostasis, thereby significantly impacting BC proliferation and tumor development. To address this, we employed murine, IK-proficient and -deficient BC cells derived from the MMTV-PyMT mouse model in combination with pharmacological blockade of IK [24]. We studied glycolytic, as well as oxidative phosphorylation activity utilizing extracellular flux analysis and fluorescence resonance energy transfer (FRET)-based biosensors for live-cell imaging of glucose, lactate, and ATP homeostasis in MMTV-PyMT-derived BC cells. In addition, we assessed whether the absence of IK affected overall glucose uptake, sub-/cellular Ca^2+^ and K^+^ homeostasis in different cellular compartments including the cytosol, endoplasmic reticulum (ER) and mitochondria. Finally, the energy-sensing kinase AMPK, a central regulator of metabolism and autophagy, as well as the regulation of autophagy-related proteins such as microtubule-associated protein 1A/1B light chain 3B (LC3B) [27], the autophagy receptor p62 [28], and Unc-51 like autophagy activating kinase 1 (ULK1) [29] were studied under different nutrient conditions. In summary, loss or pharmacologic inhibition of IK impaired Ca^2+^ and K^+^ signals in multiple cell compartments and reduced overall cancer cell metabolic activity, which in turn triggered a compensatory increase in autophagic flux rates in murine BC cells.

## Materials and methods

### BC cell model and cell culture

Cells were obtained from tumors of mouse mammary tumor virus polyoma middle T antigen (MMTV-PyMT) transgenic FVB/N mice expressing IK (WT) or with IK channel depletion (IK KO) [24, 25]. Tumor growth in vivo and biopsies were authorized by the local ethics *Committee for Animal Research* (Regierungspräsidium Tübingen, Germany) and were performed in accordance with the *German Animal Welfare Act*. The animals were kept in standardized cages on a 12 h light/dark-cycle, and the housing conditions were temperature- and humidity-controlled. We ensured that the animals had ad libitum access to food (Altromin, Lage, Germany; Sniff Spezialdiäten GmbH, Soeast, Germany) and water. Cells analyzed in this study were isolated from *n* = 5 different female breast tumor-bearing mice per genotype (~12–14 weeks of age). Upon dissection, tumors were carefully minced into pieces using atraumatic forceps, lysed by 1 mg/ml Collagenase D (Roche, Basel, Switzerland) for 10 min and cultured as indicated afterwards. Cells derived from MMTV-PyMT tumors were grown in IMEM supplemented with 5% FBS and 100 U ml^−1^ penicillin and 100 µg ml^−1^ streptomycin at 37 °C and 5% CO_2_ as described previously [24, 25]. Removal of contaminating fibroblasts was achieved by brief exposure (<1 min) of the cultures to Trypsin (2.5%) (Thermo Fisher Scientific, Waltham, USA) diluted 1:10 in PBS (final concentration of 0.25% Trypsin). Upon gently tapping the plate, detached fibroblasts were aspirated using a 10 ml serological stripette, while >90% of the BC cells remained attached during this step. Similar morphology and homogeneity of cells obtained from different tumors and mice was routinely controlled by bright-field microscopy (Fig. S1A). Upon fibroblast removal, BC cells were detached by re-incubation with 1 ml Trypsin (2.5%) (Thermo Fisher Scientific, Waltham, USA) diluted 1:10 in PBS (final concentration of 0.25% Trypsin) for 5 min in the incubator. Additionally human BC cell line MCF-7 was cultivated in DMEM (Thermo Fisher Scientific, Waltham, USA) supplemented with 10% FBS and 100 U ml^−1^ penicillin and 100 µg ml^−1^ streptomycin at 37 °C and 5% CO_2_ [23]. All cell culture materials were purchased from Corning (New York, USA), media and supplements described above from Invitrogen (Thermo Fisher Scientific, Waltham, USA). Transfection with IK rescue plasmids (MC200643, Origene, Rockville, USA) for 16 h at 37 °C and 5% CO_2_ was performed using PolyJet transfection reagent (SignaGen laboratories, Ballenger Creek, USA) according to the manufacturer’s instructions.

### Cell viability assay

MMTV-PyMT BC cells were seeded in a 96-well plate and incubated in either 0, 1, 2, or 5 µM Tram-34 in complemented IMEM (5% FBS, 1x P/S) for 48 h. To assess cell viability, 3-(4,5-Dimethylthiazol-2-yl)-2,5-Diphenyltetrazolium Bromide (MTT, ThermoFisher Scientific, Waltham, USA) was used according to manufacturer’s instructions. After the assay, absorbance was measured at 570 nm using a plate reader (Tecan infinite F200 Pro, Tecan, Männedorf, Switzerland).

### SDS-PAGE and western blot

MMTV-PyMT BC cells were either treated with 2 µM Tram-34, or equivalent amounts of DMSO and cultivated for 48 h. Prior cell lysis using lysis buffer containing (in mM): 150 NaCl, 10 NaPP, 10 NaF, 50 HEPES, 2 Na_3_VO_4_, 1 EDTA, and 1% TritonX-100 (all Carl Roth, Karlsruhe, Germany) and one tablet of cOmpleteMiniEDTAfree protease inhibitors (Merck, USA), cells were either treated with 100 nM Bafilomycin (Merck, Darmstadt, Germany) in IMEM, EBSS (Earle’s Balanced Salt Solution, Sigma Aldrich, St. Louis, USA), or combination of both for 3 h at 37 °C, 5% CO_2_. EBSS was used to study nutrient starvation (autophagy induction), whereas Bafilomycin was expected to block V-ATPase-dependent fusion and acidification during autolysosome assembly. As a result, LC3B levels were elevated under all three conditions (EBSS, Bafilomycin, EBSS + Bafilomycin). Comparison to control conditions reveals either a possible block of degradation or an accelerated flux of autophagy. The protocol to investigate the autophagic flux was adapted from previous protocols [30–32]. After 30 min incubation on ice, lysates were centrifuged for 15 min at 12,000 rpm at 4 °C. The supernatant was quantified via Bradford and adjusted to a final concentration of 0.5 to 2 µg/µl (15–25 µl per well in SDS-PAGE). For LC3B and αTubulin analysis, gradient gels (separation 16%, spacer 10%, stacking 4%, Polyacrylamid (PA) provided by Carl Roth, Karlsruhe, Germany), for every other protein 11.5% PA-gels were used. For SDS-PAGE, electrophoresis buffer was used containing (in mM): 248 Tris-HCl, 1920 glycine, and 1% of SDS, pH = 8.3 (all Carl Roth, Karlsruhe, Germany). Upon electrophoresis, semi dry transfer, PVDF-FL membranes (Merck, Darmstadt, Germany) were used at 80 mA for 1 h and 150 mA for 15 min each. For proper protein-transfer Tris-Glycin-transfer-buffers were used as a gradient of anode 1-, anode 2- and cathode-buffer containing (in mM): anode 1 (300 Tris-HCl, 20% Methanol, pH = 10.4), anode 2 (30 Tris-HCl, 20% Methanol, pH = 10.4), cathode (25 Tris-HCl, 44.2 6-Aminocaproic acid, 20% Methanol, pH = 7.6), all provided by Carl Roth (Karlsruhe, Germany). After blocking of the membranes for 1 h in 5% milk powder or BSA in 1xTBST (Carl Roth, Karlsruhe, Germany), they were washed in 1xTBST for 5 min. 1xTBST contained (in mM): 10 Tris-HCl, 140 NaCl, 0.005% Tween 20^®^ (Carl Roth, Karlsruhe, Germany), pH = 8,0). Blots were incubated at 4 °C overnight with the appropriate primary antibodies against LC3B (NB600-1384, NovusBiologicals, Littleton, USA), p62/SQSTM1 (PM045, MBL International, Woburn, USA), ULK1 (D8H5, CST, Danvers, USA), pULK1 (80218-1-RR, Proteintech, Manchester, UK), AMPK (23A3, CST, Danvers, USA), pAMPK (40H9, CST, Danvers, USA), AKT (9272, CST, Danvers, USA), pAKT (4060, CST, Danvers, USA), STK11/LKB1 (10746-1-AP, Proteintech, Manchester, UK), pLKB1 (C67A3, CST, Danvers, USA), αTubulin (DM1A, CST, Danvers, USA) and GAPDH (14C10, CST, Danvers, USA). All primary antibodies were diluted 1:1,000 in 5% BSA (Carl Roth, Karlsruhe, Germany) in 1xTBST with 0.05% NaN_3_ (Carl Roth, Karlsruhe, Germany). Following 3 washing steps, secondary antibodies conjugated to Cy5 (29038278, Amersham, Buckinghamshire, UK) or Cy3 (29038275, Amersham, Buckinghamshire, UK) and diluted 1:2,500 in 1xTBST were incubated for 1 h at room temperature. The detection of bound antibodies was visualized via fluorescence and quantified in Image Quant TL software (7.0, GE Healthcare, Chicago, USA). αTubulin and GAPDH were used as loading controls. Western Blot was modified and performed according to established protocol [24]. Full and uncropped Western blots are presented in the supplemental material file.

### IF staining of BC cells

MMTV-PyMT BC cells or human BC cell line MCF-7 were fixed using 4% PFA for 10 min. According to previously established, but slightly modified, protocols [23, 24], cells were washed twice with PBS and blocked for 1 h at RT with 10% NDS (normal donkey serum, Jackson ImmunoResearch, West Grove, USA) containing 0.1% Triton X-100 (Carl Roth, Karlsruhe, Germany) in PBS for blockage. Primary antibodies were diluted in 1.5% NDS in PBS and incubated overnight in a humidity chamber at 4 °C. Cells were then washed twice with PBS and incubated for 2 h in a dark, moisty chamber at RT with secondary antibody solution. A second incubation with a conjugated phalloidin-647 antibody (1:2,500; A22287, Thermo Fisher Scientific, Waltham, USA) in 1.5% NDS in PBS for 20 min was performed to determine cell size. Cells were covered with Perma-Fluor mounting medium (Richard-Allan Scientific, Kalamazoo, USA) with DAPI (1 µg/ml, Thermo Fisher Scientific, Waltham, USA). To visualize the antigen-antibody complexes an *Apotome* operated by Zeiss ZEN imaging software (3.0 blue edition) was used. Primary antibodies were ERα (MC-20, sc-542, Santa Cruz Biotechnology, Dallas, USA), Ki-67 (9129 S, Cell Signaling Technologies, Danvers, USA), p62 (PM045, MBL International, Woburn, USA) and KCNN4 (60276-1-Ig, Proteintech, Manchester, UK) as well as secondary antibodies AF555 (anti rabbit, Thermo Fisher Scientific, Waltham, USA) or AF488 (anti mouse, Thermo Fisher Scientific, Waltham, USA). p62 puncta per cell were automatically calculated by the software CellProfiler (4.2, CellProfiler) [31]. ERα and Ki-67 were co-stained with DAPI to determine the Ki-67 labeling-index by normalizing the number of Ki-67 positive cells to the absolute number of DAPI positive cells per image.

### FRET-based live-cell imaging

MMTV-PyMT tumor cells were seeded on 30 mm circular glass coverslips. Polyjet-transfection protocol (SignaGen laboratories, Ballenger Creek, USA) was carried out according to the manufacturer’s instructions for 16 h overnight resulting in a reproducible expression level of the FRET sensors used (General Protocol for Transfecting Mammalian Cell with PolyJet™ In Vitro DNA Transfection Reagent). Following plasmids were provided by Addgene (Watertown, USA) and transfected with Polyjet: *4mtD3cpv* (Addgene #58184), *AKTAR* (Addgene #61624), *AMPKAR* (Addgene #35097), *CARGeco1* (Addgene #45493), *cyto lc-LysM GEPII 1.0, D1ER* (Addgene #36325), *Laconic* (Addgene #44238), *mito lc-LysM GEPII 1.0, mtAT1.03*, *TORCAR*, *NesAT1.03*, *pH-Lemon – LC3B* and *Pyronic* (Addgene #51308). For imaging, glass dishes were mounted in a PC30 perfusion chamber (NGFI GmbH, Graz, Austria) and cells were preincubated for 30 min in imaging-buffer (IB). IB contained (in mM): 140 NaCl, 5 KCl, 2 CaCl_2_, 1 MgCl_2_, 10 HEPES, 10 D-glucose, 0.1% glutamine and 0.1% essential amino acids, pH adjusted to 7.4 with NaOH. All chemicals were provided by Carl Roth (Karlsruhe, Germany) or Thermo Fisher Scientific (Waltham, USA). To obtain a Ca^2+^ free buffer, the addition of CaCl_2_ was omitted and 1 mM of EGTA (Carl Roth, Karlsruhe, Germany) was added. For glucose-free conditions, D-glucose was replaced by *N*-methyl-d-glucamine (NMDG, Carl Roth, Karlsruhe, Germany). Following solutions were dissolved in various IB constellations, to induce changes in sensor-activation. The stock solutions of the compounds were prepared using DMSO and the compounds were used at the following final concentration: 3 µM BHQ (Sigma Aldrich, St. Louis, USA), 3 µM Ionomycin (Fermentek, Jerusalem, Israel), 3 µM Oligomycin-A (ChemCruz, Dallas, USA), 3 µM Gramicidin (Sigma Aldrich, St. Louis, USA), 0.5 µM FCCP (ChemCruz, Dallas, USA), 3 µM BAY-8002 (Sigma Aldrich, St. Louis, USA), 3 mM 2-DG (ChemCruz, Dallas, USA) and 100 µM ATP (Carl Roth, Karlsruhe, Germany). For pharmacological IK modulation, cells were pre-incubated in IMEM (supplemented with 5% FBS and 1x P/S) containing 2 µM Tram-34 for 48 h, and cells were maintained in IB containing Tram-34 (2 µM) during measurements.

Live cell imaging was performed on a Zeiss microscope (Zeiss Observer Z.1, Wetzlar, Germany) connected to an external light source (2200114 LED-Hub, Omicron Laserage, Rodgau-Dudendorf, Germany) according to previous investigations [3]. Microscope was equipped with a Plan-Neofluar 40x/1,30 Oil immersion objective. An Optosplit II (Cairn Research, Faversham, UK) was used for FRET-based measurements. The LED-Hub (Omicron Laserage, Rodgau-Dudendorf, Germany) equipped with a 340 nm, 380 nm, 455 nm, 470 nm and 505-600 nm LED, with 340x, 380x, 427/10, 473/10 and 575/15 bandpass filters, respectively (all AHF Analysentechnik, Tübingen, Germany), Emissions were captured using a 409/493/573/652 dichroic and 514/605/730 emission filter or a 459/526/596 dichroic with a 475/543/702 emission filter (AHF Analysentechnik, Tübingen, Germany). Images were captured using a pco.panda 4.2 bi sCMOS camera (pco., Kelheim, Germany). For image acquisition and microscope control, VisiView software (Visitron Systems, Puchheim, Germany) was used. During image acquisition, buffers were exchanged using a gravity-based perfusion system (NGFI GmbH, Graz, Austria).

### Fluorescence probes for Ca^2+^, mitochondrial membrane potential, and glucose uptake

For Ca^2+^ imaging, BC cells were seeded and measured as described before (*FRET-based live-cell imaging*) [24]. Fura-2 (AAT Bioquest, Sunnyvale, USA) loading was performed using 3.3 µM FURA-2 AM in IMEM for 60 min at 37 °C in a humidified incubator with 5% CO_2_. Followed by two washing steps in IB, emission upon excitation at 340 nm (Ca^2+^ bound to Fura-2) and 380 nm (Ca^2+^ free) were detected using Zeiss setup, without split view (described above). Changes in buffer- conditions over time, were performed as indicated by using a gravity-based perfusion system (NGFI GmbH, Graz, Austria).

For investigating mitochondrial membrane potential, Tetramethylrhodamine methyl ester (TMRM, Thermo Fisher Scientific, Waltham, USA) loading was performed (modified protocol of [33]). 200 nM of TMRM, a concentration that did not induce any toxic effects in MMTV-PyMT BC cells, was applied in IB for at least 30 min prior to live-imaging at 575/15 nm using the microscope setup described before. During imaging, TMRM remained present in all buffers and FCCP (carbonyl cyanide-p-trifluoromethoxyphenylhydrazone, ChemCruz, Dallas, USA) was added manually to a final concentration of 0.5 µM. For quantification, ratio of mitochondrial intensity to corresponding nuclei was measured over time.

Glucose uptake was assessed using fluorescent glucose analog 2-NBDG (2-deoxy-2-[(7-nitro-2,1,3-benzoxadiazol-4-yl)amino]-D-glucose, BioGems, Westlake Village, USA) [34]. In all, 100 µM 2-NBDG was added to IMEM (5% FCS, 1x P/S) and cells were incubated overnight at 37 °C, 5% CO_2_. Ensuing cells were washed in glucose-free IB, as previous described. Glucose uptake was measured at an excitation of 473/10 nm at the Zeiss Observer Z.1.

### Extracellular flux analysis

Extracellular acidification rate (ECAR) and oxygen consumption rate (OCR) were measured using a seahorse XF24 Analyzer (Agilent Technologies, USA) and carried out according to the manufacturer’s instructions. 50,000 MMTV-PyMT BC cells were seeded per well of the 24-well plates and treated either with DMSO or 2 µM Tram-34 for 24 h and during seahorse measurement. The following compounds were injected to receive the final concentrations of: 2 µM Oligomycin-A (ChemCruz, Dallas, USA), 0.2 µM FCCP (ChemCruz, Dallas, USA) and 2.5 µM Antimycin A (Sigma Aldrich, St. Louis, USA). Seahorse data was corrected for blank and was normalized to the protein concentration per well, which was assessed by BCA-assay (Thermo Scientific, Waltham, USA). OCR calculations under conditions using different inhibitors of the respiratory chain and F_0_F_1_ ATP synthase followed manufacturer’s recommendations (Agilent Technologies, Inc., Santa Clara, USA) and previously published protocols [35–37]. Survey of OCR calculations was as follows: (I) Basal OCR is measured as ratio of untreated (first 4 timepoints) to Antimycin-A treated (last 3 timepoints). (II) Ratio of Oligomycin-A induced OCR to basal level (I) reveals ATP production in the mitochondria. (III) Maximal OCR level reflects maximal respiration induced by FCCP through uncoupling of OxPhos and compensatory elevation of O_2_-consumption. (IV) Ratio of maximal (III) to baseline (I) reveals possible spare capacity of ATP-production in mitochondria. (V) Ratio of Antimycin-A induced minima of OCR to basal respiration (I) indicates the proton leakage from intermembrane space back to matrix of mitochondria.

### RNA analysis

MMTV-PyMT BC cells were cultivated on 100 mm cell culture dishes (Corning, Karlsruhe, Germany) and RNA was isolated using peqGOLD RNA pure system (PEQLab, Wilmington, USA) according to the manufacturer’s instructions. Genomic DNA contaminations were removed by a DNase digestion using 5 µl DNase (Roche, Basel, Switzerland) and 6 µl DEPC-treated water (Carl Roth, Karlsruhe, Germany) per 50 µl sample solution. Solution was incubated 30 min at 37 °C followed by inactivation of enzymes by 5 min at 80 °C. mRNA samples were adjusted to a final concentration of 0.1 µg/µl. In all, 5 µl mRNA sample were mixed with 4 µl iScript^TM^ (Bio Rad, Hercules, USA), 10 µl DEPC-water (Carl Roth, Karlsruhe, Germany) and either no or 1 µl reverse transcriptase (Bio Rad, Hercules, USA), for cDNA transcription. Followed by 30 min incubation at 42 °C and heat shock inactivation for 1 min at 95 °C, prior to addition of 180 µl DEPC-water (Carl Roth, Karlsruhe, Germany) to cDNA solution (final 200 µl). For RT-qPCR analysis samples were prepared with SsoAdvanced Universal (SYBR® Green Supermix, Bio Rad, Hercules, USA) according to the manufacturer’s instructions. Triplicates of SYBR-green fluorescence probes were detected using Opticon^TM^ (MJ Research, Saint-Bruno, Canada). Samples (Ct-) values were normalized on Ct of housekeeping genes (ACTB) and calculated as 2^-Ct (normalized)^ afterwards. All mRNA expression analysis of primary cell cultures were processed using the following primer pairs purchased from Eurofins (Luxemburg, Luxemburg): ACTB^for^ 5′-CAT TGC TGA CAG GAT GCA GAA GG-3′, ACTB^rev^ 5′-TGC TGG AAG GTG GAC AGT GAG G-3′; AMPKα^for^ 5′-GGT GTA CGG AAG GCA AAA TGG C-3′, AMPKα^rev^ 5′-CAG GAT TCT TCC TTC GTA CAC GC-3′, AMPKβ2^for^ 5′-GAC TTC GTT GCC ATC CTG GAT C-3′, AMPKβ2^rev^ 5′-CCA AGC TGA CTG GTA ACC ACA G-3′, AMPKγ1^for^ 5′-TCT CCG CCT TAC CTG TAG TGG A-3′, AMPKγ1^rev^ 5′-GCA GGG CTT TTG TCA CAG ACA C-3´, CaMMK2^for^ 5′-CAA CGT GGT GAA GCT GGT AGA G-3′, CaMMK2^rev^ 5′-TGG TCT TCG GAC AGT GGC TTG A-3′. RNA analysis was modified and performed according to established protocol [23, 38].

### Statistics

Statistical analysis was performed using GraphPad Prism software (v8.0.2.2) and provided as mean ±standard error of mean (SEM). Data were tested with a two-tailed unpaired t-test of a gaussian distribution. Comparison of >2 data sets was done either using One-way- or Two-way ANOVA followed by Tukey’s multiple comparison (MC). If variances differed between the groups, Welch´s correction was used. The statistical tests are explained in the respective figure legends. *p*-values of ≤0.05 were indicated with *, *p* ≤ 0.01 with **, and *p* ≤ 0.001 with ***, reflecting comparison of genotypes. For the comparison of treated to non-treated conditions significant differences are represented by *p* ≤ 0.05 with ^#^, *p* ≤ 0.01 with ^##^, and *p* ≤ 0.001 with ^###^. n-numbers in figure legends reflect biological replicates. MMTV-PyMT BC cells in this study derived from *n* = 5 tumor-bearing animals per genotype.

### Supplemental material

Supplemental material includes 5 figures with additional background information about the MMTV-PyMT BC cells and their pharmacological treatment using Tram-34 (or genetically rescue) on glycolytic (Fig. S1) and metabolic activity (Fig. S2). Supplemental Fig. S3 displays the pharmacological effect of Tram-34 on Ca^2+^ homeostasis in IK-proficient and -deficient BC cells, as well as changes in K^+^ level in various compartments of these cells. Down- and upstream kinases of AMPK, as well influence of Tram-34 on AMPK activity are shown in Fig. S4. Figure S5 describes the autophagic flux in MMTV-PyMT cells and the Tram-34 sensitivity of the autophagy pathway. In addition, full and uncropped Western blots are presented in the supplemental material file.

## Results

### Depletion of IK reduces glycolytic activity and capacity of primary MMTV-PyMT BC cells

**Fig. 1:**
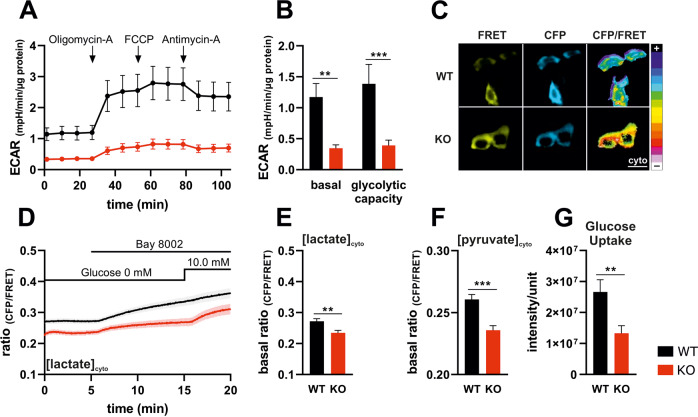
IK deficiency impairs glycolytic activity. **A** Extracellular acidification rates (ECAR) of MMTV-PyMT WT (black line and circles) and MMTV-PyMT IK KO cells (red line and circles) over-time in response to administration of Oligomycin-A, FCCP, or Antimycin-A as indicated in the panel. Data represents average ±SEM of *n* = 3 independent experiments per genotype. **B** Basal ECAR (timepoints 0–30, left) and glycolytic capacity (right) of MMTV-PyMT WT (black bars) and MMTV-PyMT IK KO cells (red bars). Bars represent average ±SEM, *n* = 3 with **p ≤ 0.01 and ***p ≤ 0.001, unpaired *t*-test. **C** Representative FRET (yellow, left), CFP (cyan, middle) and pseudocoloured FRET-ratio images (16-colors, right) and **D** FRET-ratio signals over-time of MMTV-PyMT WT (black) and MMTV-PyMT IK KO cells (red) expressing *Laconic*, a FRET-based lactate indicator ([lactate]_cyto_). At time point indicated in the panel, either the MCT-inhibitor BAY-8002 or 10.0 mM of glucose were administered to the cells. Data represents average ±SEM, *n* = 5. Scalebar = 20 µm. **E** Basal FRET-ratio signals (timepoints 0–5 in **D**) of MMTV-PyMT WT (black bar) and MMTV-PyMT IK KO cells (red bar) expressing the FRET-based lactate indicator. Bars represent average ±SEM, *n* = 5 with **p ≤ 0.01, unpaired *t*-test. **F** Basal FRET-ratio signals (timepoints 0–5) of MMTV-PyMT WT (black bar) and MMTV-PyMT IK KO cells (red bar) expressing *Pyronic*, a FRET-based pyruvate indicator ([pyruvate]_cyto_). Bars represent average ±SEM, *n* = 5 (WT), 6 (KO) with ***p ≤ 0.001, unpaired t-test. **G** Glucose uptake rates of MMTV-PyMT WT (black bar) and IK KO cells (red bar), analyzed using 2-NBDG, a fluorescent glucose analog. Data represent average ±SEM from *n* = 5 independent experiments per genotype.

To address a putative link between energy metabolism and IK expression, we utilized primary murine BC cells either derived from WT or IK KO FVB/N mice of the tumor-prone MMTV-PyMT model. Using these cells, we first confirmed the previously identified role of endogenous IK channels for cell proliferation by determining the Ki-67 index and their Her2 (data not shown) and ERα positivity in comparison to the established human BC cell model MCF-7 (Fig. S1B–D) [24]. Next, we assessed extracellular acidification rates (ECAR) i.e., lactate secretion, of murine MMTV-PyMT WT and IK KO cells via extracellular flux analysis in real-time (Fig. 1A, B). Compared to MMTV-PyMT WT cells, these experiments unveiled a significant decrease in basal ECAR levels of IK KO cells at all timepoints (Fig. 1A, B). Subsequently, Oligomycin-A (2 µM), a blocker of F_0_F_1_ ATP synthase, FCCP (0.2 µM), an uncoupler of the respiratory chain, and Antimycin-A (2.5 µM), a complex III inhibitor, were administered to measure ECAR at different respiratory states. In addition to the lower basal glycolytic activity, these inhibitors of mitochondrial respiration revealed a reduced glycolytic capacity in BC cells lacking IK (Fig. 1A, B).

To verify these findings, we next aimed to quantify intracellular lactate concentrations ([lactate] _cyto_), as a direct correlation of ECAR [39]. Therefore, MMTV-PyMT WT and MMTV-PyMT IK KO cells were transfected with a FRET-based lactate sensor (Fig. 1C) [40]. In the absence of glucose [lactate]_cyto_ levels were lower in IK KO *versus* WT cells (Fig. 1C–E). Subsequent blockage of the monocarboxylate transporter 1 (MCT1) by BAY-8002 increased cytosolic [lactate]_cyto_ by reducing the levels of lactate secretion. As MCT1 mediates the co-transport of lactate with a proton (H^+^), this finding may partially explain the proportional increase in extracellular acidification i.e., H^+^ accumulation, in MMTV-PyMT-derived WT BC cells (Fig. 1A, B) [41]. Re-administration of glucose to the BC cells initiated a second increase in [lactate]_cyto_ of both genotypes, reflecting the full functionality of the FRET-based biosensor in both cell types, and confirming cellular lactate production from glycolysis (Fig. 1D). To further verify that the lack of IK diminishes the glycolytic activity, a FRET-based pyruvate biosensor was employed [42]. In the absence of IK, cytosolic pyruvate concentrations ([pyruvate]_cyto_) estimated with this biosensor, were lower compared to control cells (Fig. 1F). To address whether the [pyruvate]_cyto_ depletion relies on changes in glucose availability, we assessed cellular glucose uptake rates using the fluorescent glucose analog 2-NBDG. These experiments showed that glucose uptake rates were significantly impaired in IK negative BC cells (Fig. 1G), again confirming a previously unknown contribution of IK channels to the accelerated glucose transport and metabolism in BC cells as discussed later in more detail [43–46].

To further substantiate a link between IK and the cellular energy metabolism, we used a pharmacological approach for IK inhibition. To elucidate whether triarylmethane-34 (Tram-34), a specific IK blocker, influenced the viability of MMTV-PyMT WT and IK KO BC cells, an MTT assay was performed. Importantly, the viability of MMTV-PyMT BC cells was not significantly affected by Tram-34 independent of the cell´s IK status (Fig. S1A). Subsequent analysis of ECAR revealed a clear tendency to reduce ECAR values in Tram-34 treated MMTV-PyMT WT cells compared to untreated cells, while IK KO cells remained unaffected by Tram-34 (Fig. S1C, D). Accordingly, FRET imaging exhibited a drop of [lactate]_cyto_ in Tram-34 exposed WT cells to the level observed in IK KO cells (Fig. S1E). These experiments indicate that both an acute pharmacological inhibition as well as the chronic ablation of the IK gene impact the overall glycolytic activity of MMTV-PyMT BC cells.

### IK deficiency alters mitochondrial ATP homeostasis and metabolism

**Fig. 2:**
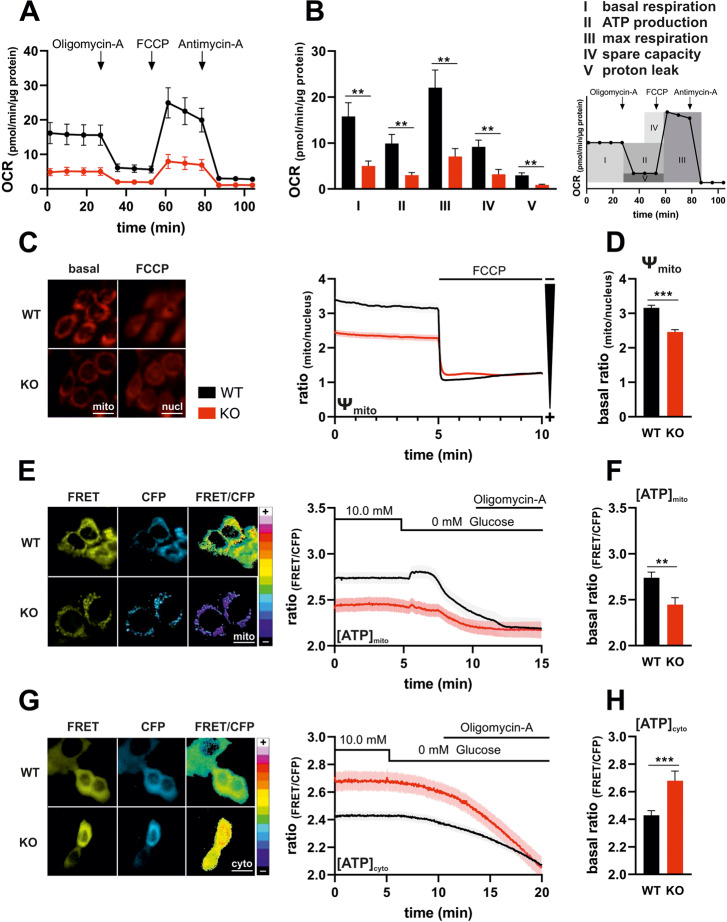
IK deficiency alters mitochondrial ATP homeostasis and metabolism. **A** Oxygen consumption rate (OCR) of MMTV-PyMT WT (black line and circles) and MMTV-PyMT IK KO cells (red line and circles) over-time in response to administration of Oligomycin-A, FCCP, or Antimycin-A as indicated in the panel. Data represents average ±SEM of *n* = 3 independent experiments per genotype. **B** Basal OCR (I: timepoints 0–30 in **A**), ratio of Oligomycin-A induced OCR to basal level (II), maximal respiration induced by FCCP through uncoupling of OxPhos (III), ratio of maxima to baseline (IV), and ratio of Antimycin-A induced minima to basal respiration (V) of MMTV-PyMT WT (black bars) and MMTV-PyMT IK KO cells (red bars). Bars represent average ±SEM, *n* = 3 with **p ≤ 0.001, unpaired *t*-test. **C** Representative images (left panel) and fluorescence ratio over-time of mitochondrial/nuclear fluorescence (right panel) of MMTV-PyMT WT (left panel, upper images, and right panel, black line) and MMTV-PyMT IK KO cells (left panel, lower images, and right panel, red line), either under basal conditions (left panel, basal) or upon administration of FCCP for mitochondrial uncoupling (left panel, FCCP) at time point indicated in the right panel. Data represent average ±SEM from *n* = 9 independent experiments. Scalebar = 20 µm. **D** Corresponding basal Ψ_mito_ values (timepoints 0–5) of curves as shown in (C) with ***p ≤ 0.001, unpaired *t*-test. **E** Representative FRET (left panel, yellow, left), CFP (left panel, cyan, middle) and pseudocoloured FRET-ratio images (left panel, 16-colors, right) and FRET-ratio signals over-time (right panel) of MMTV-PyMT WT (black) and MMTV-PyMT IK KO cells (red) expressing *mtAT1.03*, a FRET-based mitochondrial ATP indicator ([ATP]_mito_). At time point indicated in the panel, either glucose was removed, or Oligomycin-A was administered to the cells. Data represents average ±SEM, *n* = 8. Scalebar = 20 µm. **F** Corresponding basal mitochondrial ATP levels (timepoints 0–5) of curves as shown in **E** with **p ≤ 0.01, unpaired *t*-test. **G** Representative FRET (left panel, yellow, left), CFP (left panel, cyan, middle) and pseudocoloured FRET-ratio images (left panel, 16-colors, right) and FRET-ratio signals over-time (right panel) of MMTV-PyMT WT (black) and MMTV-PyMT IK KO cells (red) expressing *NesAT1.03*, a FRET-based ATP indicator targeted to the cytosol ([ATP]_cyto_). At time point indicated in the panel, either glucose was removed, or Oligomycin-A was administered to the cells. Data represents average ±SEM, *n* = 5. Scalebar = 20 µm. **H** Corresponding basal cytosolic ATP levels (timepoints 0–5) of curves as shown in **G** with **p ≤ 0.01, unpaired *t*-test.

As a result of modified glycolytic activity under IK depletion and pharmacological inhibition, we next assessed the oxygen consumption rates (OCR) of MMTV-PyMT WT and IK KO cells as a measure of mitochondrial bioenergetics. Again, and in accordance with the ECAR rates (Fig. 1A, B), this comparison exposed a reduced basal OCR of IK KO cells (Fig. 2A, B I). As expected, OCRs in both genotypes were sensitive to ATP synthase inhibition by Oligomycin-A (Fig. 2A, B). However, the difference between basal to Oligomycin-A treated OCR levels, indicative of the actual mitochondrial ATP production [35, 47], was significantly decreased in MMTV-PyMT cells lacking IK (Fig. 2B II). To trigger maximal OCR, FCCP, an uncoupling agent that destroys the H^+^ gradient across the inner mitochondrial membrane, was injected. Under this condition, lack of IK was associated with lower maximal respiration (Fig. 2A, B III) as well as reduced spare capacity, constituting the difference between basal respiration and respiration at its maximal level as robust functional parameter of the mitochondrial reserve (Fig. 2A, B IV). Finally, administration of Antimycin-A for complex III inhibition unveiled that the H^+^ leak, representing the movement of H^+^ back into the mitochondrial matrix in an F_0_F_1_ ATP-synthase independent manner, was reduced in MMTV-PyMT IK KO cells (Fig. 2A, B V). In summary, the OCR profiles of MMTV-PYMT BC cells provide strong evidence for a reduced mitochondrial metabolic activity and capacity in the absence of functional IK channels, which is also confirmed by the pharmacological blockade of IK with Tram-34, resulting in impaired OCR rates (Fig. S2A, B).

Next, we assessed mitochondrial membrane potential (ΔΨ_mito_) as a possible reason for diminished ATP production in MMTV-PyMT IK KO cells. Therefore, cells were loaded with tetramethylrhodamin-methylester (TMRM), which accumulates in negatively charged mitochondria, thereby indicating the current ΔΨ_mito_, while background sequestration to the nucleus was used as a control readout (Fig. 2C). TMRM mitochondria-to-nucleus fluorescence ratio indicated a more polarized ΔΨ_mito_ in WT compared to KO cells. Importantly, by interfering with the H^+^ gradient FCCP depolarized the mitochondrial membrane and thereby equalized the difference in TMRM signals between genotypes (Fig. 2C, D). The less polarized ΔΨ_mito_ was also observed in Tram-34 treated MMTV-PyMT WT cells, whereas in the absence of IK ΔΨ_mito_ remained unaffected by Tram-34 (Fig. S2C, D). Vice versa, we re-transfected the channel back into the IK KO background (IK rescue). This approach did not alter the viability of the cells (Fig. S1A) but increased basal ΔΨ_mito_ polarization to values seen in IK-proficient WT BC cells (Fig. S2C, D). To further verify the successful IK channel rescue, immunofluorescence staining of transfected and non-transfected MMTV-PyMT IK KO cells was performed (Fig. S1B). These experiments confirmed a predominant plasma membrane staining for IK in IK rescue BC cells, while IK-deficient cells remained IK negative as expected (Fig. S1B, lower panel and merge).

Subsequently, we reasoned that altered OCR and ΔΨ_mito_ would impact mitochondrial ATP ([ATP]_mito_) production. To assess this a FRET-based, mitochondrial matrix targeted ATP biosensor was employed in MMTV-PyMT WT and IK KO cells [48]. Transient transfection of the cells resulted in mitochondrial localization of the sensor in both genotypes (Fig. 2E). In line with previous results, we observed higher basal [ATP]_mito_ in IK-proficient cells (Fig. 2E, F). Importantly, glucose removal followed by administration of Oligomycin-A resulted in blockage of cellular ATP production and decreased [ATP]_mito_ to the same level in both genotypes (Fig. 2E). Moreover, the monitoring of FRET ratio signals in the presence of Tram-34 revealed that basal [ATP]_mito_ of MMTV-PyMT WT, but not IK KO cells, was sensitive to pharmacological IK channel inhibition (Fig. S2E, F). Next, we examined, whether the lower metabolic activity also resulted in reduced cytosolic [ATP] concentration ([ATP]_cyto_). MMTV-PyMT cells expressing the FRET-based ATP biosensor in the cytoplasm [48] were measured over time (Fig. 2G) at baseline, followed by glucose removal, and Oligomycin-A treatment (Fig. 2E). As observed for [ATP]_mito_, blockage of mitochondrial ATP production decreased [ATP]_cyto_ irrespectively of the IK status of the BC cells to the same level (Fig. 2G). In contrast to the lower basal [ATP]_mito_ in IK depleted BC cells, assessment of basal [ATP]_cyto_ revealed significantly higher levels for IK-deficient *versus* IK-proficient BC cells (Fig. 2H).

In summary, these experiments indicated an altered metabolic activity of IK depleted or pharmacologically inhibited cells showing both, discrete glycolytic (Fig. 1) and mitochondrial metabolic alterations (Fig. 2). These decreases in glycolytic and mitochondrial bioenergetics are counteracted by a higher [ATP]_cyto_ in the KO (Fig. 2H).

### Depletion of IK alters sub-/cellular Ca^2+^ homeostasis

**Fig. 3:**
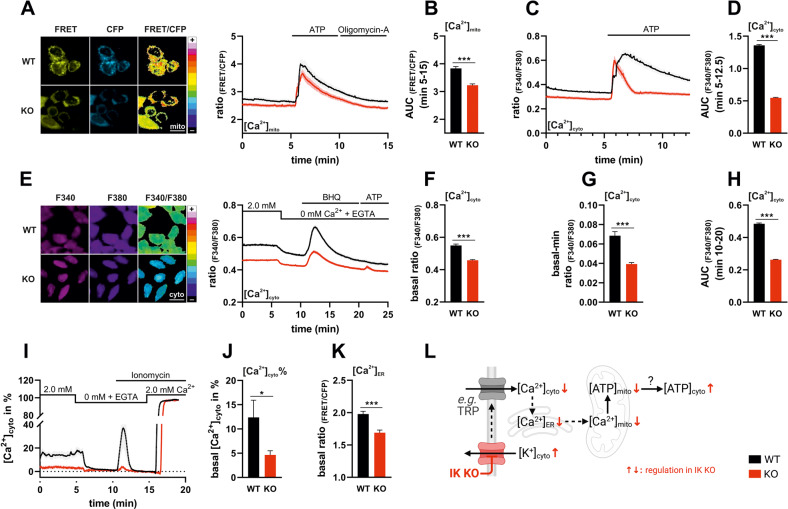
Loss of IK impacts subcellular Ca^2+^ homeostasis. **A** Representative FRET (left panel, yellow, left), CFP (left panel, cyan, middle), and pseudocoloured FRET-ratio images (left panel, 16-colors, right) and FRET-ratio signals over-time (right panel) of MMTV-PyMT WT (black) and MMTV-PyMT IK KO cells (red) expressing *4mtD3cpv*, a FRET-based mitochondrial Ca^2+^ indicator ([Ca^2+^]_mito_). At time point indicated in the panel, either extracellular ATP, to trigger release of Ca^2+^ from intracellular stores via metabotropic receptors, or Oligomycin-A for inhibition of ATP synthase were administered. Scalebar = 20 µm. **B** Area under the curve (AUC) from timepoints 5–15 min of FRET-ratio signals as shown in **A**. Data represents average ±SEM, *n* = 6 with ***p ≤ 0.001, unpaired *t*-test with Welch’s correction for different varriances. **C** Fluorescence emission ratio signals of MMTV-PyMT WT (black line) and MMTV-PyMT IK KO cells (red line) loaded with FURA-2 over-time in response to administration of extracellular ATP at time point indicated in the panel. Data represents average ±SEM, *n* = 6 independent experiments per genotype. **D** Area under the curve (AUC) from timepoints 5–12.5 min of FRET-ratio signals as shown in **C** represent average ±SEM, *n* = 6 with ***p ≤ 0.001, unpaired *t*-test with Welch’s correction for different varriances. **E** Representative images of MMTV-PyMT WT (left panel, upper lane, and right panel, black curve) and MMTV-PyMT IK KO cells (left panel, lower lane, and right panel red curve) loaded with FURA-2, either at an excitation of 340 nm (left panel, pink, left) or 380 nm (left panel, violette, middle). Right images show pseudocoloured ratio image (left panel, 16-colors, right). FURA-2-ratio signals were recorded over-time (right panel) of MMTV-PyMT WT (black) and MMTV-PyMT IK KO cells (red) in response to extracellular removal of Ca^2+^ (0 mM Ca^2+^ + EGTA), administration of BHQ for SERCA inhibition, or cell stimulation with ATP at indicated timepoints. Data represents average ±SEM of *n* = 6 independent experiments per genotype. Scalebar = 20 µm. In **F** basal FURA-2 ratio signals (timepoints 0–5 in **E**), **G** the difference of basal- to the minimal FURA-2 ratios of timepoints 5–10 min and **H** area under the curve (AUC) from timepoints 10–20 min of MMTV-PyMT WT (black bars) and MMTV-PyMT IK KO cells (red bars) are shown. Data represent means ± SEM of *n* = 6 with ***p ≤ 0.001, unpaired *t*-test with Welch’s correction for different varriances. **I** Normalized fluorescence over time signals of MMTV-PyMT WT (black line) and MMTV-PyMT IK KO cells (red line) expressing *Car-GECO1*, a single FP-based, red fluorescent Ca^2+^ sensor located in the cytosol ([Ca^2+^]_cyto_). At time points indicated in the panel, extracellular Ca^2+^ was removed (0 mM Ca^2+^ + EGTA), Ionomycin was added and extracellular Ca^2+^ (2.0 mM) was re-added. Data represents average ±SEM of *n* = 5 independent experiments per genotype. **J** Basal Ca^2+^ MMTV-PyMT WT (black bar) and MMTV-PyMT IK KO cells (red bar) expressing *Car-GECO1* (timepoints 0–5 in **I**). Bars represent average ±SEM, *n* = 5 with *p ≤ 0.05, unpaired *t*-test. **K** Basal FRET-ratio signals (timepoints 0–5) of MMTV-PyMT WT (black bar) and MMTV-PyMT IK KO cells (red bar) expressing *D1ER* ([Ca^2+^]_ER_), a FRET-based Ca^2+^ indicator targeted to the endoplasmic reticulum (ER). Data represents average ±SEM of *n* = 3 with ***p ≤ 0.001, unpaired *t*-test. **L** Putative consequences of IK channel deficiency on Ca^2+^ signaling pathways in MMTV-PyMT BC cells. Figure created using BioRender.

Impaired metabolism contrasts a higher [ATP]_cyto_, this prompted us to assess the cellular Ca^2+^ homeostasis of MMTV-PyMT WT and IK KO cells. Since IK channels are both, Ca^2+^-activated and involved in regulation of the Ca^2+^ homeostasis [49], we hypothesized that, among other compartments, Ca^2+^ levels in the mitochondrial matrix might be altered, which in turn would affect ATP production [50–52]. Accordingly, the K^+^ influx into the matrix is considered as major contributor to the mitochondrial membrane potential (∆ψ_mito_) regulating cellular respiration [53]. In this regard, IK contributed to OxPhos in pancreatic carcinoma cells [54], and mitoIK channels, among other K^+^ “uniporters” in the mitochondrial system, might directly serve to control the metabolism of these organelles [55]. Hence, the genetically encoded mitochondrial matrix targeted Ca^2+^ sensitive indicator *4mtD3cpV* was used to transiently transfect MMTV-PyMT BC cells [56]. Cells lacking IK showed a reduction of mitochondrial Ca^2+^ concentration ([Ca^2+^]_mito_) over time in response to extracellular ATP, acting on purinergic receptors (Fig. 3A, B) [57, 58]. Elucidating the cytosolic Ca^2+^ concentrations ([Ca^2+^]_cyto_) using FURA-2, a significant decrease of basal [Ca^2+^]_cyto_ was observed in IK KO cells (Fig. 3C, F). Again, upon extracellular ATP administration, MMTV-PyMT IK KO cells revealed lower [Ca^2+^]_cyto_ over time (Fig. 3C, D). These experiments may point to an altered Ca^2+^ handling of IK KO cells affecting multiple cellular compartments including the lumen of the endoplasmic reticulum (ER). To verify these findings, we next examined [Ca^2+^]_cyto_ upon removal of extracellular Ca^2+^ (0 mM) using additionally EGTA as a Ca^2+^ chelator (Fig. 3E). With the administration of the Ca^2+^-free buffer, a drop of [Ca^2+^]_cyto_ was observed in both genotypes, albeit the response was more pronounced in MMTV-PyMT WT compared to MMTV-PyMT IK KO cells (Fig. 3E, G). These results emphasize, that the extracellular Ca^2+^ concentration ([Ca^2+^]_ex_) is important for MMTV-PyMT cells to maintain their [Ca^2+^]_cyto_ homeostasis. Further, ER calcium ([Ca^2+^]_ER_) was depleted by applying 2,5-Di-t-butyl-1,4-benzohydroquinone (BHQ), a frequently used inhibitor of the sarcoplasmic/endoplasmic reticulum Ca^2+^ ATPase (SERCA), which evoked [Ca^2+^]_cyto_ elevations, most probably due to blocking Ca^2+^ re-entry from the cytosol into the ER [59]. Interestingly, BHQ provoked [Ca^2+^]_cyto_ elevation estimated by AUC analysis, was less pronounced in IK KO compared to WT cells (Fig. 3E, H), again pointing to a lower [Ca^2+^]_ER_ content under these conditions. Ultimately, administration of extracellular ATP confirmed a full [Ca^2+^]_ER_ depletion (Fig. 3E). To ensure that all effects were indeed caused by the IK channel, MMTV-PyMT cells were treated with Tram-34, which abolished the difference in [Ca^2+^]_cyto_ dynamics between WT and IK KO cells (Fig. S3A–C). To validate these findings and to further investigate how IK channels affect BC Ca^2+^ signaling, we next utilized *Car-GECO1* a genetically encoded, intensiometric indicator for recording [Ca^2+^]_cyto_ (Fig. 3I, J) [60] and *D1ER* a FRET-based Ca^2+^ biosensor targeted to the lumen of the ER (Fig. 3K) [61]. Normalization of the fluorescence intensity to minimal and maximal fluorescence intensities upon cell treatment with the Ca^2+^ ionophore ionomycin in the presence (2.0 mM Ca^2+^) or absence of Ca^2+^ (0 mM Ca^2+^, EGTA) (Fig. 3I) confirmed the difference of basal [Ca^2+^]_cyto_ between WT and IK KO MMTV-PyMT cells (Fig. 3J). Together, BHQ- (Fig. 3E) and ionomycin- based experiments (Fig. 3I), suggest a diminished storage capacity of [Ca^2+^]_ER_ in IK KO cells. To confirm this assumption, we assessed basal [Ca^2+^]_ER_, representing the major Ca^2+^ store in the cell [62], using *D1ER*. In the absence of IK these experiments unveiled significantly decreased levels of [Ca^2+^]_ER_ (Fig. 3K), confirming an altered global and sub-/cellular Ca^2+^ homeostasis (Fig. 3E–J).

We next elucidated whether subcellular K^+^ concentrations ([K^+^]) were altered in an IK-dependent manner as K^+^ homeostasis could influence Ca^2+^ fluxes [18, 63] and simultaneously represents a link to ATP production [3, 64, 65]. BC cells were transfected with genetically encoded FRET-based K^+^ indicator (lc-LysM GEPII 1.0), either targeted to the cytosol or mitochondrial matrix, respectively [66]. Interestingly, mitochondrial [K^+^] ([K^+^]_mito_) was significantly elevated in MMTV-PyMT IK KO cells (Fig. S3D, E), albeit [K^+^]_mito_ remained virtually unaffected upon triggering intracellular Ca^2+^ elevations evoked by cell stimulation with extracellular ATP in both genotypes. The responsiveness of the mitochondrial-targeted mito lc-LysM GEPII 1.0 is demonstrated by cell treatment with gramicidin, an ionophoric peptide, leading to a drop of [K^+^]_mito_ in both genotypes and equalized [K^+^]_mito_ at a minimum (Fig. S3 D). Analogous differences as determined in [K^+^]_mito_ were observed for cytosolic [K^+^] ([K^+^]_cyto_), as [K^+^]_cyto_ was elevated in MMTV-PyMT IK KO *versus* WT BC cells (Fig. S3F, G). Upon gramicidin administration, the drop of [K^+^]_mito_ follows with some delay to the [K^+^]_cyto_, suggesting an interdependence of [K^+^] across these subcellular compartments (Fig. S3D, F).

This disturbance in subcellular [K^+^] and [Ca^2+^] in various cellular compartments could explain the diminished metabolic activity of IK depleted cells (Fig. 3L). It remains unclear, however, if and mechanistically how these changes in ion composition account for the increase in [ATP]_cyto_.

### Lack of IK impairs cell metabolism and elevates AMPK activity

**Fig. 4:**
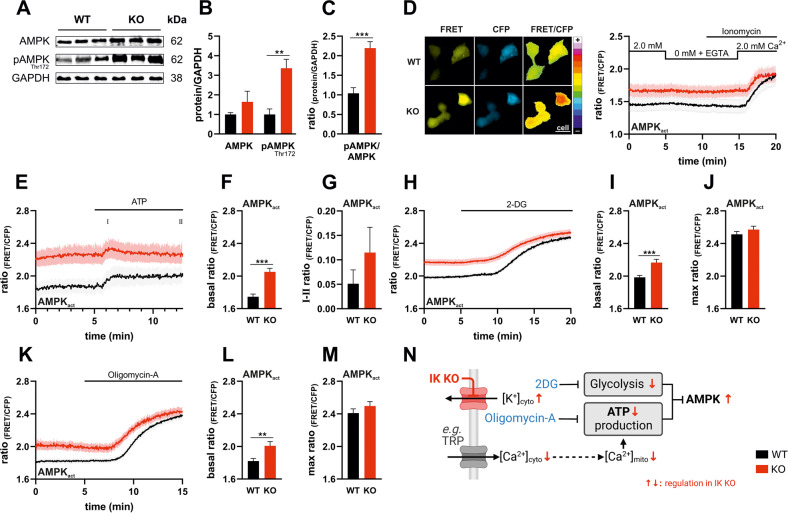
IK KO induced changes in metabolism and Ca^2+^ elevate AMPK activity. **A** Western blot analysis of GAPDH, AMPK, and phosphorylated AMPK at Thr^172^ in protein lysates obtained from MMTV-PyMT WT (left) and MMTV-PyMT IK KO (right) cells. Data represents average ±SEM of *n* = 6 independent experiments per genotype. **B** Quantification of Western blot band intensities as seen in **A** and normalization on GAPDH of MMTV-PyMT WT (black bar) and MMTV-PyMT IK KO (red bar), respectively. Bars represent average ±SEM, *n* = 6 with **p ≤ 0.01, unpaired *t*-test. **C** Demonstrates ratio of phosphorylated AMPK to whole AMPK intensities normalized on GAPDH of MMTV-PyMT WT (black bar) and MMTV-PyMT IK KO (red bar). Bars represent average ±SEM, *n* = 6 with ***p ≤ 0.001, unpaired *t*-test. **D** Representative FRET (left panel, yellow, left), CFP (left panel, cyan, middle), and pseudocoloured FRET-ratio images (left panel, 16-colors, right) and FRET-ratio signals over-time (right panel) of MMTV-PyMT WT (black) and MMTV-PyMT IK KO cells (red) expressing *AMPKAR*, a FRET-based AMPK activity reporter (AMPK_act_). Extracellular removal of Ca^2+^ (0 mM Ca^2+^ + EGTA), administration of Ionomycin, or Ca^2+^ (2.0 mM) re-addition were performed as indicated in the panel. Data represents average ±SEM of *n* = 5 independent experiments per genotype. Scalebar = 20 µm. **E** FRET-ratio signals over time of MMTV-PyMT WT (black line) and MMTV-PyMT IK KO cells (red line) expressing *AMPKAR*. Administration of extracellular ATP, i.e., triggering of intracellular Ca^2+^ release, reveals changes in AMPK activity at indicated timepoints. Data represents average ±SEM of *n* = 5 independent experiments per genotype. **F** Basal FRET-ratio values (timepoints 0–5) and **G** the difference of maxima- (I) to the endpoint (II) FRET-ratios of MMTV-PyMT WT (black bars) and MMTV-PyMT IK KO cells (red bars), of curves as shown in **E**. Bars represent average ±SEM, *n* = 5 with ***p ≤ 0.001, unpaired *t*-test. **H** FRET-ratio signals over time of MMTV-PyMT WT (black line) and MMTV-PyMT IK KO cells (red line) expressing AMPKAR. Administration of glycolysis inhibitor 2-DG reveals changes in AMPK activity at indicated timepoints. Data represents average ±SEM of *n* = 6 independent experiments per genotype. **I** Basal (timepoints 0–5) and **J** maximal FRET-ratio signals of MMTV-PyMT WT (black bars) and MMTV-PyMT IK KO cells (red bars) expressing *AMPKAR*. **I**, **J** demonstrate corresponding FRET-ratio signals of curves shown in **H**, either under basal conditions (timepoints 0–5 in (**I**)) or after treatment with 2-DG (**J**). Bars represent average ±SEM, *n* = 6 with ***p ≤ 0.001, unpaired *t*-test with Welch’s correction for different varriances. **K** FRET-ratio signals of MMTV-PyMT WT (black line) and MMTV-PyMT IK KO cells (red line) expressing *AMPKAR* over time. Administration of ATP synthase inhibitor Oligomycin-A reveals changes in AMPK activity at indicated timepoints. Data represents average ±SEM of *n* = 6 independent experiments per genotype. **L** Basal (timepoints 0–5) and **M** maximal FRET-ratio signals of *AMPKAR* expressing MMTV-PyMT WT (black bars) and MMTV-PyMT IK KO cells (red bars) of curves as demonstrated in **K**. Bars represent average ±SEM, *n* = 6 with **p ≤ 0.01, unpaired *t*-test. **N** Summarizing scheme of the influence of IK channel deficiency (red arrows) on energy yield and AMPK activation. Figure created using BioRender.

We reasoned that the maintenance of a high [ATP]_cyto_ would induce genotype-specific differences in AMPK activity. AMPK represents an energy-sensing kinase, activated by either an increase in AMP and/or ADP relative to ATP, or Ca^2+^[11]. AMPK can compensate for energy fluctuations and, hence, reflects cytosolic energy homeostasis [11]. First, we investigated the expression levels of AMPK and the phospho-AMPK (pAMPK) level at Thr^172^, which, in addition to the allosteric activation by AMP, is indicative of AMPK activation. Total AMPK was not different between MMTV-PyMT WT compared to IK KO cells, while pAMPK levels were significantly higher in protein lysates derived from IK KO cells (Fig. 4A–C). To functionally validate the immunoblot-based findings in living cells, we transfected a FRET-based AMPK activity reporter, *AMPKAR*, and investigated AMPK activity (AMPK_act_) in WT compared to IK KO cells (Fig. 4D) [67]. Basal FRET-ratio signals received from the two genotypes confirmed the results of an increased AMPK activity in IK KO cells (Fig. 4D). While IK channel inhibition using Tram-34 increased AMPK_act_ to levels present in IK KO (Fig. S4A), IK rescue diminished activation to the same extent as observed in MMTV-PyMT WT cells, clearly emphasizing IK channel-specific effects on AMPK (Fig. S4A). To elucidate the putative link between intracellular Ca^2+^ and AMPK activity, we removed extracellular Ca^2+^ (0 mM in the presence of EGTA), followed by Ionomycin addition, and subsequently Ca^2+^ (2.0 mM) buffer administration (Fig. 4D), in analogy to earlier experiments (Fig. 3I). Surprisingly, neither MMTV-PyMT WT, nor IK KO cells demonstrated changes in AMPK activity upon cellular Ca^2+^ depletion (Fig. 4D). Re-administration of extracellular Ca^2+^, however, resulted in an increase in AMPK activity with no differences between genotypes, which confirms the expected Ca^2+^-dependent activation of AMPK in MMTV-PyMT cells (Fig. 4D). To examine whether endogenous alterations of [Ca^2+^]_cyto_ induce changes in AMPK activity we applied the purinergic receptor agonist ATP (Fig. 4E). This approach again confirmed the increased AMPK activity under basal conditions (Fig. 4E, F). Although evoking intracellular Ca^2+^ signals by ATP increased *AMPKAR* FRET ratio signals (Fig. 4E), differences in cytosolic Ca^2+^ transients between WT and IK KO (Fig. 3C, D) did not translate into alterations in AMPK activity over time (Fig. 4E, G). Accordingly, AMPK subunit analysis by qRT-PCR revealed no changes of the subunits AMPKα, AMPKβ, or AMPKγ between the two genotypes (Fig. S4B). Likewise, Ca^2+^-dependent AMPK regulators such as CaMKK2 and Calmodulin were unaltered irrespective of the cell’s IK status (Fig. S4C–E). As AMPK is activated by both, Ca^2+^ and cellular energy stress, subsequent experiments focussed on the influence of energy stress on AMPK activation. First, liver kinase B1 (LKB1), a main upstream kinase of AMPK responsible for mediating the energy-sensing was studied. LKB1 in complex with Mo25/STRAD was postulated to directly phosphorylate AMPK at Thr^172^ in response to energy limitation, although the exact mechanism is not fully clarified yet [68–70]. Overall LKB1 protein levels were not different between IK KO and WT cells, while amplified phosphorylation of LKB1 at Ser^428^ in IK depleted cell, possibly explaining AMPK activation (Fig. S4F–H). To verify that alteration in energy stress impacts AMPK activation we treated cells with the glycolysis inhibitor 2-deoxy-D-glucose (2-DG) which induced an elevation of *AMPKAR* (AMPK_act_) in WT and IK KO cells and abolished the differences in basal AMPK activity (Fig. 4H, J). A similar effect was observed upon inhibiting ATP-synthase-dependent ATP production using the F_0_F_1_ ATP synthase blocker Oligomycin-A (Fig. 4K–M). These experiments provide strong evidence that glycolysis, as well as mitochondrial ATP production, are directly involved in regulating AMPK activity in WT and IK KO cells (Fig. 4N).

### AMPK partly compensates for reduced energy metabolism by enhancing autophagy

**Fig. 5:**
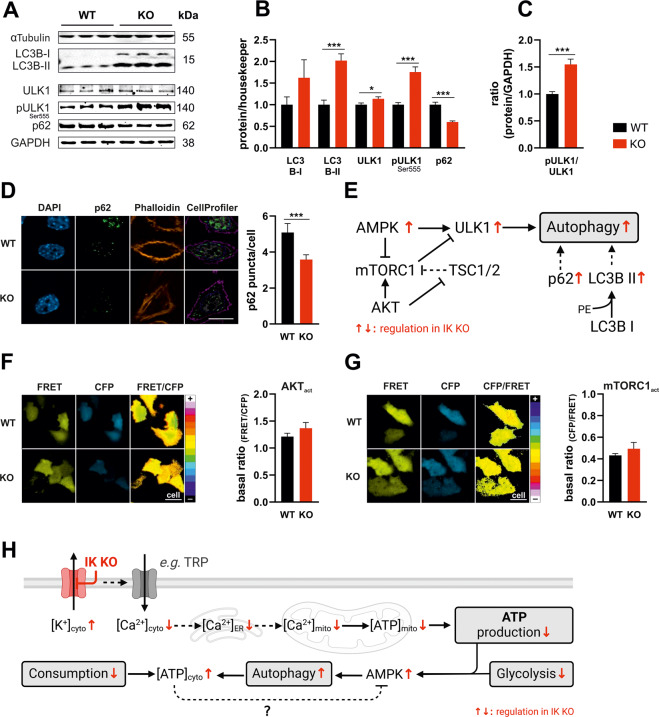
AMPK activation elevates autophagy in IK-deficient cells. **A** Western blot analysis of αTubulin, LC3B (I + II), ULK1, phosphorylated ULK1 at Ser^555^, p62 and GAPDH of protein lysates obtained from MMTV-PyMT WT (left) and MMTV-PyMT IK KO (right) cells. Data represents average ±SEM of *n* = 6 independent experiments per genotype. **B** Quantification of Western blot band intensities as demonstrated in **A** and normalization of LC3B (I + II) on αTubulin and ULK1, pULK1 and p62 on GAPDH of MMTV-PyMT WT (black bar) and MMTV-PyMT IK KO (red bar). Bars represent average ±SEM, *n* = 6 with *p ≤ 0.05 and ***p ≤ 0.001, unpaired *t*-test. **C** Ratio of phosphorylated ULK1 to total ULK1 protein normalized on GAPDH of MMTV-PyMT WT (black bar) and MMTV-PyMT IK KO (red bar). Bars represent average ±SEM, *n* = 6 with ***p ≤ 0.001, unpaired *t*-test. **D** Representative immunofluorescence (IF) staining of DAPI (left panel, blue, left), p62 (left panel, green, middle left), Phalloidin (left panel, orange, middle right) and pseudocoloured CellProfiler overlay images (left panel, merged, right) and quantification of p62 puncta per cell (right panel) of MMTV-PyMT WT (black bars) and MMTV-PyMT IK KO (red bars). Bars represent average ±SEM, *n* = 4 with ***p ≤ 0.001, unpaired *t*-test. Scalebar = 10 µm. **E** Summarizing scheme of autophagic pathway and the consequence of IK channel deficiency (red arrows). AKT: Protein kinase B; mTORC1: Mammalian target of rapamycin complex 1; TSC1/2: Tuberous sclerosis proteins 1/2. **F** Representative FRET (left panel, yellow, left), CFP (left panel, cyan, middle) and pseudocoloured FRET-ratio images (left panel, 16-colors, right) and basal FRET-ratio signals (right panel) of MMTV-PyMT WT (black bar) and MMTV-PyMT IK KO cells (red bar) expressing *AKTAR*, a FRET-based AKT indicator (AKT_act_). Bars represent average ±SEM, *n* = 5, unpaired *t*-test. Scalebar = 20 µm. **G** Representative FRET (left panel, yellow, left), CFP (left panel, cyan, middle) and pseudocoloured FRET-ratio images (left panel, 16-colors, right) and basal FRET-ratio signals (mean of 5 min measurement, right panel) of MMTV-PyMT WT (black bar) and MMTV-PyMT IK KO cells (red bar) expressing *TORCAR*, a FRET-based mTORC1 indicator (mTORC1_act_). Bars represent average ±SEM, *n* = 6, unpaired *t*-test. Scalebar = 20 µm. **H** Summarizing scheme of metabolic pathway and the influence of IK channel deficiency (red arrows). Lack of IK results in a decreased Ca^2+^ load in multiple cellular compartments affecting mitochondrial ATP-production. Consistent with lower glycolytic activity in MMTV-PyMT IK KO cells, these events trigger AMPK activation, leading to compensatory induction of autophagy. Together with the postulated lower energy consumption, autophagy explains the enhanced cytosolic ATP level in IK-depleted cells. Figure created using BioRender.

Activation of AMPK hinders anabolic-, while promoting catabolic-, energy-saving processes [71] thereby regulating autophagy. Thus, we assessed autophagic flux in MMTV-PyMT WT and KO cells, which provides energy under nutrient limiting conditions from the conversion of unused organelles, proteins, and cell components into energy-rich molecules [72–74]. Autophagic markers were studied by Western blot analysis (Fig. 5A) [73, 75]. Cytosolic LC3B, a protein that is cleaved to LC3-I and conjugated to phosphatidylethanolamine (PE) of autophagic membranes, then referred to as LC3-PE or LC3-II, is a frequently used indicator for autophagy [76]. LC3-II, which is degraded within autolysosomes [76], was increased in MMTV-PyMT IK KO cells compared to MMTV-PyMT WT cells (Fig. 5A, B). Furthermore, using fed and starved (EBSS) conditions, we administered Bafilomycin A1, a V-ATPase blocker, to prevent autolysosomal acidification and LC3-II degradation (Fig. S5A, B) in order to assess the autophagic flux in MMTV-PyMT cells [77]. As expected, blocking LC3-II degradation by Bafilomycin A1 treatment further elevated LC3-II levels in IK KO cells. This effect was even more pronounced upon cell treatment with EBSS, or a combination of Bafilomycin A1 and EBSS treatment (Fig. S5B), underscoring the increased autophagic flux in MMTV-PyMT IK KO cells [78]. Further, autophagy receptor p62 abundance, a marker for selective autophagy that becomes degraded in autolysosomes [78], was addressed by immunoblotting (Fig. 5A, B) and by *CellProfiler* analysis using immunostaining of endogenous p62 (Fig. 5D) [79]. In line with our results on LC3 lipidation, an elevated autophagic flux was indicated by significantly reduced p62 protein levels (Fig. 5A, B) and fluorescent p62 puncta (Fig. 5D) in IK KO cells when compared to WT cells.

Next, we assessed the AMPK-mediated phosphorylation of *Unc-51 like autophagy activating kinase 1* (ULK1) at Ser^555^, indicative for autophagy induction [72]. Interestingly, loss of IK provoked higher ULK1 phosphorylation levels in MMTV-PyMT cells presumably due to AMPK hyperactivation (Fig. 5A–C). To ensure that all reported effects were indeed caused by the IK channel activity, AMPK, LC3B, and p62 levels were assessed in MMTV-PyMT cells treated with Tram-34. Higher rates of autophagic flux were observed in the presence of Tram-34 (Fig. S5C, D). Moreover, using *pH-Lemon-LC3B*, a FRET-based pH indicator fused to LC3B [80], increased accumulation of LC3 in acidic autolysosomes was observed in IK KO when compared to WT MMTV-PyMT BC cells (Fig. S5E). As an alternative to the AMPK/ULK1 (Ser^555^) signaling, autophagy can also be triggered by modulation of PI3K/AKT and mTOR. Consequently, we measured AKT and mTORC1 activity levels using the FRET-based biosensors AKTAR (AKT_act_) and TORCAR (mTORC1_act_), respectively. Using these probes unveiled no difference between MMTV-PyMT WT and IK KO cells (Fig. 5F, G) [81, 82]. Accordingly, neither total AKT levels, nor phosphorylated AKT (pAKT) levels at Ser^473^ or pAKT/AKT ratios were differed between genotypes (Fig. S5F–H).

Taken together, our results emphasize that IK is involved in maintaining the cellular Ca^2+^, K^+^ and energy homeostasis and its genetic ablation or pharmacologic inhibition triggers autophagy in primary murine MMTV-PyMT BC cells (Fig. S5I).

## Discussion

Here, we present novel findings demonstrating a contribution of IK channel deficiency to BC metabolism promoting AMPK-driven autophagy. Our functional analyses of BC cells obtained from a MMTV-PyMT mouse model confirmed, that BC glycolysis, oxidative phosphorylation, Ca^2+^ homeostasis, and autophagy are dependent on the functionality of IK. This is in line with previous experiments demonstrating that IK inhibition or its global depletion in a FVB/N-based MMTV-PyMT model of BC improves survival and reduces cell proliferation rates in vitro and tumor growth in vivo [22, 24, 26, 83]. Thus, we postulate a link between these IK-dependent BC cell behaviors and unique energy production- and consumption-pathways (Fig. 5H).

Our findings may especially gain importance in cancer development and therapy due to the altered expression pattern of K^+^ channels in many cancer entities including BC [15, 16, 84]. As a result of their localization in different cellular membranes [18], K_Ca_ channels may serve as sensors for various cellular signals transmitting information between distinct cellular compartments. This will alter, for instance, the ion i.e., Ca^2+^ and K^+^ homeostasis and related signaling pathways, membrane potentials as well as sub-/cellular volume(s) [63, 85, 86]. By directly correlating K^+^- and Ca^2+^-dynamics with the metabolic activity of the cells, our study describes a possible new mechanism for targeted anti-BC therapies. This is relevant as IK channel activity contributes to BC malignancy and it can be modulated pharmacologically, which should promote further in vivo studies. Besides Tram-34 used herein for the functional analysis of acute IK modulation in multiple ex vivo BC cell “treatment” scenarios, Senicapoc, a selective inhibitor of IK, was introduced in clinical trials and was previously tested as a safe and well tolerated drug [87]. To the best of our knowledge, it was not assessed whether this clinical candidate inhibits BC development in an IK-dependent manner, while promising anti-cancer effects attributed to Senicapoc derive from cellular and pre-clinical studies on melanoma, glioma, and non-small cell lung cancer [87–90].

Our first approaches to explain the reduced tumor development in MMTV-PyMT IK KO cells [24], revealed a diminished glycolytic activity of cells lacking IK. Extracellular acidification rates, and levels of [lactate]_cyto_ and [pyruvate]_cyto_ were significantly reduced upon IK depletion and inhibition. Loss of IK significantly reduced ECAR, which could be altered due to diminished H^+^-linked lactate secretion via glycolysis, or reduced CO_2_ production by the TCA cycle [91]. To ensure that glycolysis itself is responsible for the reduced ECAR, we furthermore assessed [lactate]_cyto_ using a fluorescent biosensor and confirm lower lactate accumulation in the absence of IK. Additionally, OCRs were measured, revealing a decreased mitochondrial respiratory activity in IK KO cells as well. Using specific inhibitors of different complexes of the respiration chain, the balance from energy produced in OxPhos was shifted towards glycolysis [47]. Upon cell treatment with Oligomycin-A or FCCP, ECAR was elevated in both genotypes, but the overall effect of these compounds was tremendously reduced in IK KO cells, again confirming the reduced glycolytic rates in these cells. To explain these observations, we next assessed the glucose uptake rates and capacities of both, WT and IK KO cells. Interestingly, reduced glucose uptake was observed in MMTV-PyMT IK KO BC cells, thereby explaining the lower rates of glycolysis, pyruvate, and lactate production. Interestingly, glucose metabolism and tolerance seem to be dependent on IK channels [44, 45]. IK expression correlated with GLUT1 expression patterns, and it was linked to sodium ion-dependent glucose transporters (SGLTs) [43, 92, 93]. Although we did not assess the expression levels of the glucose transporting proteins in MMTV-PyMT cells, future studies should consider the question if and how IK channels physically or functionally interact with different glucose “transporting” mechanisms in BC cells. So far, our present data emphasize, however, a crucial role of IK in modulating the glycolytic activity of BC cells.

Besides the decreased glycolytic activity in IK KO cells, analysis of OCR and [ATP]_mito_ measurements also revealed an altered mitochondrial energy production. As seen in pancreatic ductal adenocarcinoma by Kovalenko et al. [54], a knockdown of IK resulted in a decrease in OCR. Usage of FCCP as mitochondrial uncoupler to receive maximal OCRs revealed lower maximal O_2_ consumption rates of IK depleted cells. To explain the reduced energy production, ΔΨ_mito_ measurements were performed, ultimately representing the driving force for H^+^ to re-enter the mitochondrial matrix and finally yielding ATP production [94]. Indeed, loss of IK reduced ΔΨ_mito_, explaining disturbed mitochondrial energy production. In line with the previous findings of lower glycolysis rates and, hence, fewer educts for the TCA cycle, these results are congruent with the reduced mitochondrial ATP production. In addition, an interplay of the ΔΨ_mito_ generated by the electron transport chain and the mitochondrial Ca^2+^ uptake was postulated [95]. Along these lines, elevated matrix Ca^2+^ levels directly influence mitochondria-dependent energy production [50, 52], and IK channel activation, in turn, is also dependent on Ca^2+^ [96]. To investigate, whether altered Ca^2+^ homeostasis between WT and IK KO BC cells might be involved in mitochondrial ATP production, subcellular Ca^2+^ imaging experiments were performed. These experiments unveiled, that a loss of IK leads to overall lower [Ca^2+^] in multiple cell compartments including the cytosol, the mitochondrial matrix (AUC) as well as the lumen of the ER. We postulate that these changes in Ca^2+^ ensue from largely impaired cellular K^+^ fluxes, as we find an accumulation of [K^+^]_cyto_ and [K^+^]_mito_ in IK KO cells. Because our genetical approach does not allow us to discriminate between differentially localized (endogenous) IK channels, we cannot rule out that the effects on [K^+^] dynamics resulted from changes in mitoIK activity. It would require modulators specifically targeting mitoIK to assess the impact of these channels directly, but such compounds are at present not available. However, upon application of the K^+^ ionophore gramicidin, the drop in FRET/CFP ratio in the [K^+^]_cyto_ compartment preceded the drop in [K^+^]_mito_ (dashed line in Fig. S3D, F). The resulting delay in time possibly shows the direct influence of [K^+^]_cyto_ on [K^+^]_mito_, albeit this again does not exclude additional effects of gramicidin on the IMM or a contribution of mitoIK, which allows K^+^ flux into the mitochondrial matrix. Regarding IK’s role in regulating the intracellular Ca^2+^ concentration, it is widely accepted that the channel promotes the Ca^2+^ uptake across the plasma membrane [97, 98], which couples changes in membrane potential to the duration, amplitude and shape of Ca^2+^ signaling events in different subcellular compartments. Accordingly, after [Ca^2+^]_ER_ depletion, the store-operated Ca^2+^ entry (SOCE) was also dependent on IK’s activity [99, 100]. Thus, conversely, depletion of the IK channel would explain both, a lower [Ca^2+^]_cyto_ and lower [Ca^2+^]_ER_. As described earlier by Rizzuto et al. [101], also the proximity of the ER, a compartment with extremely high storage capacity for Ca^2+^, to mitochondria facilitates the Ca^2+^ transfer into the mitochondrial matrix [102]. Through mitochondria-associated membranes of the ER, the Ca^2+^ release from this compartment is closely connected to the outer mitochondrial membrane [103]. Subsequently we hypothesize that in IK KO cells the reduced [Ca^2+^]_cyto_ and diminished [Ca^2+^]_ER_ provided less driving force for the mitochondrial Ca^2+^ uptake, resulting in diminished Ca^2+^-dependent production of mitochondrial ATP [50, 52].

Reduced glycolytic and mitochondrial activity is known to activate AMPK [71], as cells constantly adapt their energy homeostasis. As a result of metabolic stress, AMPK triggers catabolic- and inhibits anabolic pathways [71, 104]. Due to the reduced energy homeostasis observed in IK depleted cells, AMPK activity, as indicated by phosphorylation at Thr^172^, was increased. A direct correlation of glycolytic or mitochondrial ATP production to AMPK activity in MMTV-PyMT cells was seen upon cell treatment with 2-DG or Oligomycin-A. The attained decrease in ATP levels resulted in significantly higher AMPK activity levels in IK WT and IK KO cells with no differences in the maximal AMPK activity between genotypes upon energy stress induction. This implies that at least the acute response of AMPK to metabolic changes is intact in IK-deficient BC cells and presumably dependent on AMP/ADP ratios [71].

Although our study revealed IK as major player in modulating BC cell metabolism, AMPK activation and autophagy, some important questions remained open. Indeed, it seems counterintuitive that lower [ATP]_mito_, a lower rate of glycolysis and less ATP production via OxPhos result in higher [ATP]_cyto_ in the IK KO. Based on the data obtained, we reasoned that autophagy-mediated nutrient recycling and energy conservation as well as a coincident reduction in energy consumption by IK KO, also reflected by a lower proliferation rate of IK-deficient cells (Fig. S1), could explain this apparent discrepancy (Fig. 5H). Both processes lead to an elevated [ATP]_cyto_, while we did not determine resulting consequences on the ratios of AMP/ATP or ADP/ATP. The effect on the ratios might be, however, important as small changes in AMP or ADP concentration can activate AMPK despite high [ATP]_cyto_ [105].

Due to these highly dynamic changes in AMPK activity, changes in [ATP]_cyto_ without the influence of AMPK, which contributes to intracellular ATP recovery, are difficult to investigate. Additionally, the actual inducer of AMPK activity at the γ-subunit are either AMP or ADP, which are difficult to monitor in real-time in living cells [11]. Besides, an AMP/ADP-independent regulatory mechanism for AMPK activity via the glycolytic substance fructose-1,6-bisphosphate on LKB1 was postulated [106]. LKB1 directly phosphorylates and activates AMPK [71, 106]. In the IK KO higher LKB1 phosphorylation levels were observed, and this may, in turn, promote kinase activity causing an AMP/ADP-independent increase in AMPK activity.

In general, activation of AMPK in MMTV-PyMT IK KO cells may serve as a compensatory mechanism promoting energy production due to insufficient metabolic activity. Besides triggering catabolic processes, AMPK activity, which non-canonically is also regulated by mitochondrial ROS (ROS_mito_), was shown to promote mitochondrial fission, mitophagy, CREB- and PGC-1α-dependent transcription as well as autophagic processes [71, 107]. In our study, we focused on a putative link between the cell’s IK status, AMPK activation, and the autophagy pathway, so we cannot exclude differences in, for instance, mitochondrial quantity, ROS_mito_ production or CREB- and PGC-1α-regulated gene expression [107, 108].

AKT exerts inhibitory effects on autophagy that are mediated by activation of mTORC1, but both factors were unaltered in the absence of IK [6, 7]. Thus, we hypothesized, that AMPK directly phosphorylates ULK1 to induce the formation of the autophagy-initiating ULK1 complex [72]. In addition to the higher pULK1 levels, key autophagy markers downstream were affected in MMTV-PyMT IK KO cells. Accordingly, the autophagic degradation activity, determined in the presence and absence of lysosomal inhibitors and/or starvation media, was elevated according to LC3-II, digested p62 and decreased autolysosomal pH levels. This confirms higher autophagic flux in IK KO cells. Since autophagy generates, for instance, fatty acids and amino acids, which can be catabolized to yield energy, this energy delivering process could explain the elevated [ATP]_cyto_ in the absence of IK [5, 71]. Nevertheless, further investigations are required to clarify the exact mechanism underlying the high levels of AMPK activity despite high [ATP]_cyto_ in IK KO cells.

Taken together, our study reveals a crucial involvement of IK K^+^ channels in regulating cellular K^+^ and Ca^2+^ homeostasis, which are important to maintaining glucose uptake, glycolysis, and [ATP]_mito_ production (Fig. 5H). These findings are in line with the very recently identified role of K^+^ for hexokinase II activity. This glycolytic enzyme catalyzes and accelerates glycolysis, thereby providing a link between K^+^ channel-mediated K^+^ signals and the metabolic program of tumor cells [3]. Because of the observed energy constraints in IK KO cells, autophagy, providing substrates for both, de novo biosynthesis and energy generation, is induced in an AMPK/ULK1-dependent manner. However, this mechanism is not able to fully compensate for the high glycolytic activity and energy requirements, as MMTV-PyMT BC cell proliferation and tumorigenesis are largely diminished in the absence of IK [24]. These findings suggest that pharmacological modulation of cancer-associated IK channel activity may improve the metabolic vulnerability of BC cells to support current BC therapies for the ultimate benefit of patients with cancer.

## Supplementary information

CDDIS-22-2294-checklist

Supplemental Material

## Data Availability

All datasets generated and analyzed during this study are available from the corresponding author on reasonable request.
