# Supplementary material for: IKCa channels control breast cancer metabolism including AMPK-driven autophagy
Source: Cell Death Dis. 2022 Oct 27;13(10):902. doi: 10.1038/s41419-022-05329-z (PMC9613901; doi:10.1038/s41419-022-05329-z)
Supplement: Supplementary file 2 — Supplemental Material [file 41419_2022_5329_MOESM2_ESM.docx]

**IK_Ca_ channels control breast cancer metabolism including AMPK-driven autophagy**

Dominic Gross^1^, Helmut Bischof^1^, Selina Maier^1^, Katharina Sporbeck^2^, Andreas L. Birkenfeld^3,4^, Roland Malli^5,6^, Peter Ruth^1^, Tassula Proikas-Cezanne^2^, Robert Lukowski^1,§^

^1^Department of Pharmacology, Toxicology and Clinical Pharmacology, Institute of Pharmacy, University of Tübingen, Germany

^2^Department of Molecular Biology, Interfaculty Institute of Cell Biology, University of Tübingen

^3^Institute of Diabetes Research and Metabolic Diseases (IDM), the Helmholtz Center, Munich, Germany

^4^Department of Internal Medicine IV, Division of Endocrinology, Diabetology and Nephrology, University Hospital of Tübingen, Tübingen, Germany

^5^Gottfried Schatz Research Center for Cell Signalling, Metabolism and Aging, Division of Molecular Biology and Biochemistry, Medical University of Graz, 8010 Graz, Austria

^6^BioTechMed-Graz, 8010 Graz, Austria

^§^corresponding author:

Robert Lukowski

Department of Pharmacology, Toxicology and Clinical Pharmacy

Institute of Pharmacy

University of Tübingen

Auf der Morgenstelle 8

72076 Tübingen, Germany

E-Mail: robert.lukowski@uni-tuebingen.de

Phone: +49 7071 26 74 550

Fax: +49 7071 29-2476

**Supplemental Figures and Legends:** SFig.1-5 (p.2 to p.8) plus full and uncropped Western

Blots (p.9 to p.15)


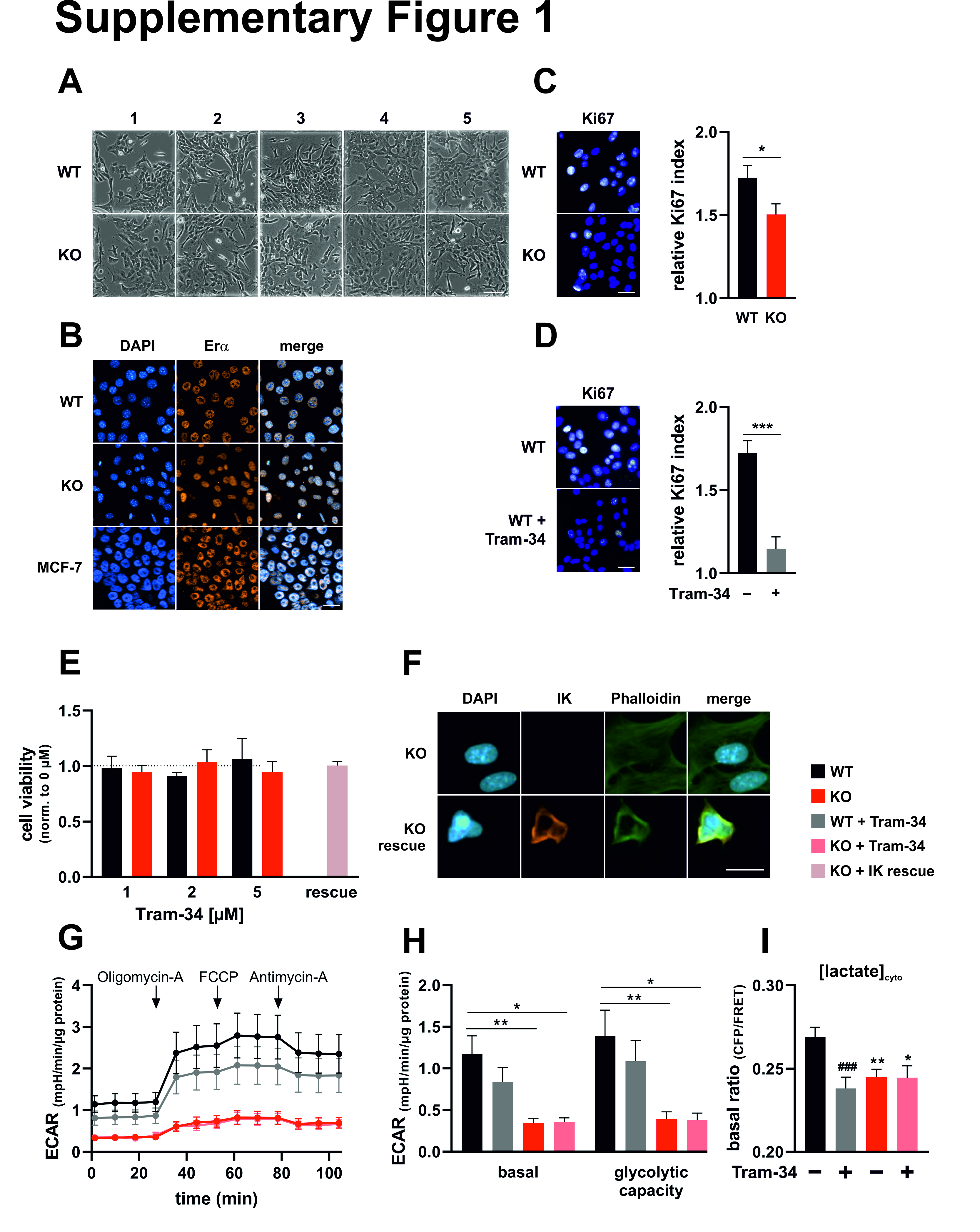


**Figure S1.** *Characterization of the MMTV-PyMT* *breast cancer model including consequences* *on glycolytic activity after genetic and pharmacological IK channel modulation.* (**A**) Brightfield images of 5 different MMTV-PyMT WT (upper panel) and IK KO BC cell cultures (lower panel) established from n = 5 different tumour bearing mice per genotype. Scalebar = 40 µm. (**B**) Representative immunofluorescence (IF) images of DAPI (blue, left), estrogenic receptor α (ERα) as a tumour marker (orange, middle) and merged images (right) of MMTV-PyMT WT (upper panel), IK KO (middle panel) and MCF-7 as positive control (lower panel), n = 3 independent experiments. Scalebar = 20 µm. (**C**) Representative merged immunofluorescence (IF) images of DAPI (blue) and proliferation marker Ki-67 (yellow) of MMTV-PyMT WT (upper left panel) and IK KO (lower left panel). Relative Ki-67-labeling index of MMTV-PyMT WT (black bar, right) and IK KO (red bar, right), normalized to total nuclei per image, n = 9 independent experiments. Scalebar = 20 µm. (**D**) Representative merged immunofluorescence (IF) images of DAPI (blue) and proliferation marker Ki-67 (yellow) of MMTV-PyMT WT (upper left panel) and WT treated with 10 µM TRAM-34 (for 24 h, lower left panel). Percentage of cells with Ki-67-positive nuclear immunostaining treated with DMSO (black bar, right) or 10 µM TRAM-34 (for 24 h, grey bar, right), n = 6 independent experiments. Scalebar = 20 µm. (**E**) Cell viability rates of MMTV-PyMT WT (black bar) and MMTV-PyMT IK KO (red bar) treated with Tram-34 for 48 hours at the indicated concentrations, or IK KO cells transfected with IK (bright red bars). Data are normalized to DMSO treated control and represents average ± SEM of n = 9 independent experiments per genotype. (**F**) Representative immunofluorescence (IF) images of DAPI (blue, left), IK channel (orange, middle left), Phalloidin (green, middle right) and merged images (merge, right) of MMTV-PyMT IK KO (upper panel) and MMTV-PyMT IK KO transfected with IK (lower panel). n = 5 independent experiments. Scalebar = 10 µm. (**G**) Extracellular acidification rates (ECAR) of MMTV-PyMT WT (black and grey line and circles), or MMTV-PyMT IK KO cells (red and salmon line and circles) over-time in response to administration of Oligomycin-A, FCCP, or Antimycin-A as indicated in the panel. Cells were either treated with DMSO (black and red) or treated with 2 µM of TRAM-34 (for 24 h, grey and salmon) Data represents average ± SEM of n = 3 independent experiments per genotype. (**H**) Basal ECAR (timepoints 0 – 30, left) and glycolytic capacity (right) of MMTV-PyMT WT (black and grey bar) and MMTV-PyMT IK KO cells (red and salmon bar) either treated with DMSO (black and red) or 2 µM TRAM-34 (for 24 h, grey and salmon). Bars represent average ± SEM, n = 3 *p≤0.05, **p≤0.01, one-way ANOVA. (**I**) Basal FRET-ratio signals (timepoints 0 – 5) of MMTV-PyMT WT (black and grey bars) and MMTV-PyMT IK KO cells (red and salmon bars) expressing *Laconic*, a FRET-based lactate indicator ([lactate]_cyto_). Cells were either treated with DMSO (black and red) or TRAM-34 (for 24 h, grey and salmon) Bars represent average ± SEM, n = 6 *p≤0.05, **p≤0.01, ^###^p≤0.001 (^#^ = compared to untreated condition of same genotype), one-way ANOVA.

**
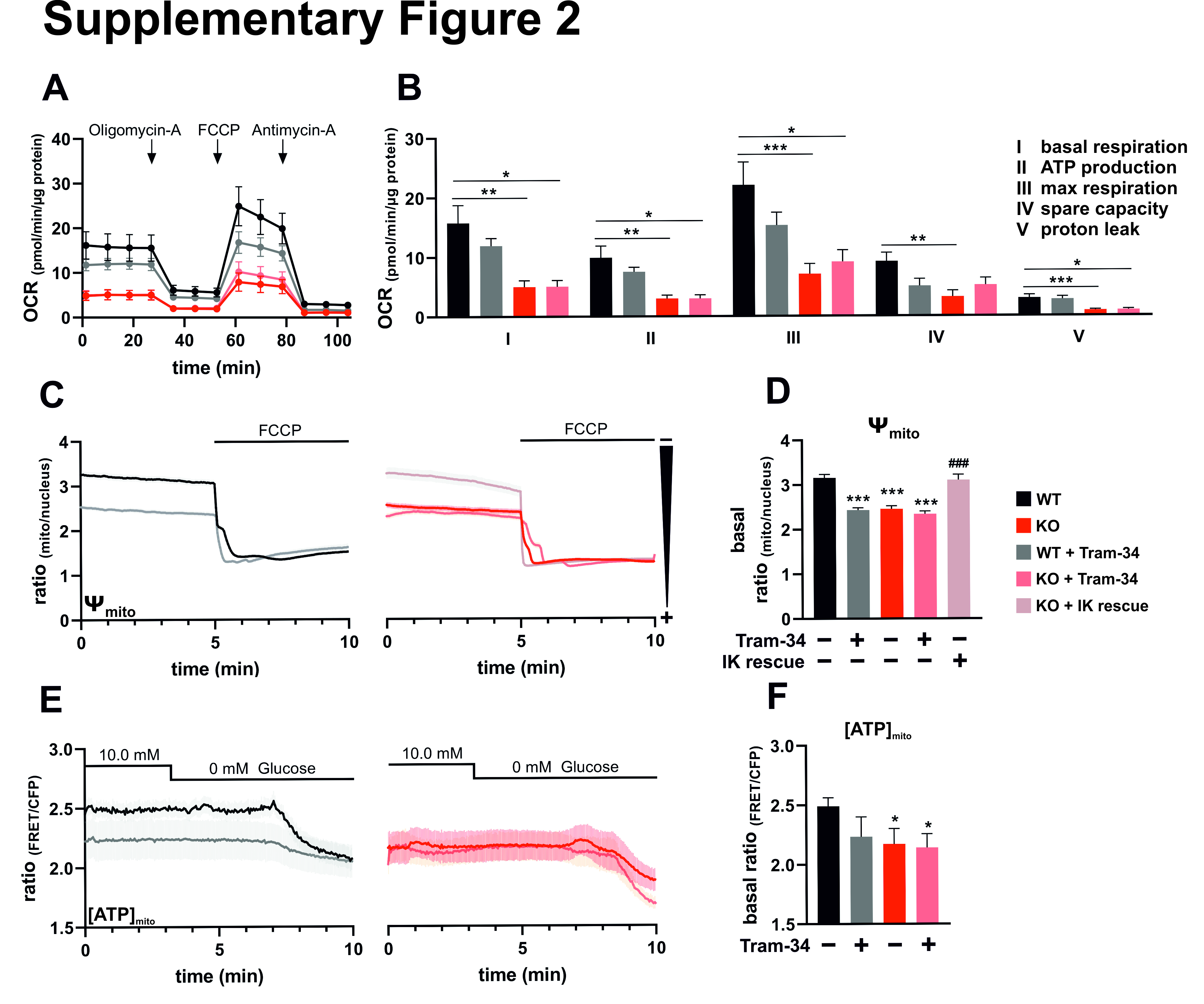
**

**Figure S2.** *Genetic and pharmacological IK channel modulation alter mitochondrial metabolism.* (**A**) Oxygen consumption rates (OCR) of MMTV-PyMT WT (black and grey line and circles), or MMTV-PyMT IK KO cells (red and salmon line and circles) over-time in response to administration of Oligomycin-A, FCCP, or Antimycin-A as indicated in the panel. Cells were either treated with DMSO (black and red) or treated with 2 µM of TRAM-34 (for 24 h, grey and salmon). Data represents average ± SEM of n = 3 independent experiments per genotype. (**B**) Basal OCR (timepoints 0 - 30, I), change of Oligomycin-A induced OCR to basal level (II), maximal respiration induced by FCCP uncoupling of OxPhos (III), change of maxima to baseline (IV), and change of Antimycin-A induced minima to basal respiration (V) of MMTV-PyMT WT (black and grey bar) and MMTV-PyMT IK KO cells (red and salmon bar) either treated with DMSO (black and red) or 2 µM TRAM-34 (for 24 h, grey and salmon). Bars represent average ± SEM, n = 3 *p≤0.05, **p≤0.01, ***p≤0.001, one-way ANOVA. (**C**) Mitochondrial membrane potential (Ψ_mito_) over-time of MMTV-PyMT WT cells either treated with DMSO (left panel, black line) or 2 µM TRAM-34 (for 48 h, left panel, grey line), or MMTV-PyMT IK KO cells treated with DMSO (right panel, red line) or 2 µM TRAM-34 (for 48 h, right panel, salmon line), or cells transiently expressing IK upon transfection (right panel, bright red line). At time point indicated in the panels, FCCP was administered to the cells for mitochondrial uncoupling. Data represent average ± SEM from n = 9 independent experiments. (**D**) Demonstrates corresponding basal Ψ_mito_ values (timepoints 0 – 5) of curves shown in (C). ***p≤0.001, ^###^p≤0.001 (^#^ = compared to untreated condition of same genotype), one-way ANOVA. (**E**) FRET-ratio signals of MMTV-PyMT WT (black and grey bar) and MMTV-PyMT IK KO cells (red and salmon bar) either treated with DMSO (black and red) or 2 µM TRAM-34 (for 48 h, grey and salmon) expressing *mtAT1.03*, a FRET-based mitochondrial ATP indicator over-time. At time point indicated in the panels, glucose was withdrawn. Data represents average ± SEM, n = 5. (**F**) Demonstrates corresponding basal mitochondrial ATP level (timepoints 0 – 5) of curves shown in (E). Bars represent average ± SEM, n = 5 *p≤0.05, one-way ANOVA.


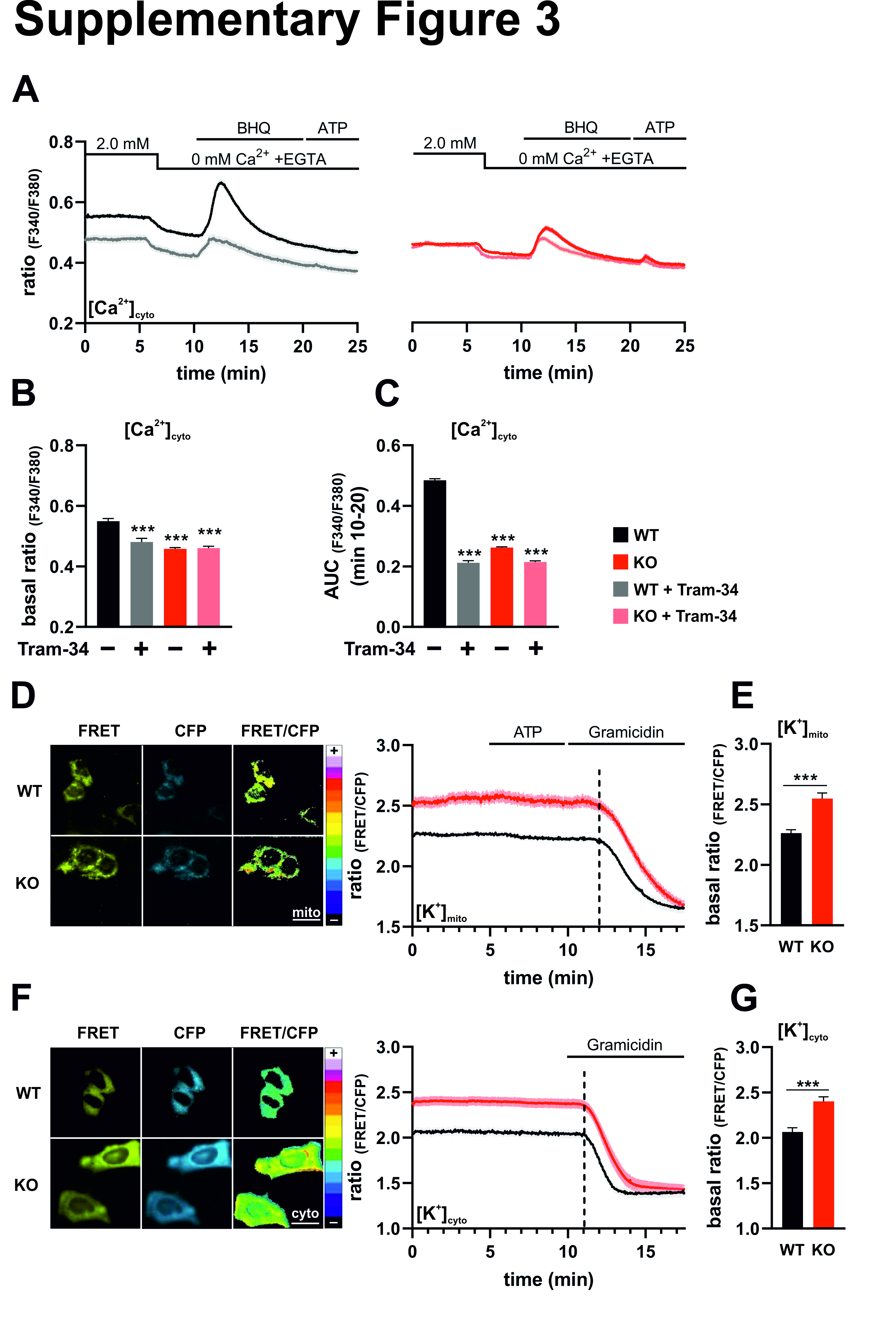


**Figure S3.** *Pharmacological IK channel inhibition impairs calcium homeostasis.* (**A**) Fluorescence emission ratio signals over-time of MMTV-PyMT WT (left panel) and MMTV-PyMT IK KO cells (right panel) loaded with FURA-2. Cells were either treated with DMSO (black and red line) or 2 µM TRAM-34 (for 48 h, grey and salmon line). At time points indicated in the panels, either extracellular Ca^2+^ was removed (0 mM Ca^2+^ +EGTA), BHQ was added for SERCA inhibition or cells were stimulated with ATP. Data represents average ± SEM of n = 6 independent experiments per genotype. (**B**) Basal FURA-2 ratios (timepoints 0 – 5) and (**C**) area under the curve (AUC) from timepoints 10 – 20 minutes of MMTV-PyMT WT and MMTV-PyMT IK KO cells either treated with DMSO or TRAM-34 as indicated. Data represent means ± SEM of n = 6 ***p≤0.001, one-way ANOVA. (**D**) Representative FRET (left panel, yellow, left), CFP (left panel, cyan, middle) and pseudocoloured FRET-ratio images (left panel, 16-colors, right) and FRET-ratio signals over-time (right panel) of MMTV-PyMT WT (black) and MMTV-PyMT IK KO cells (red) expressing *mito GEPII 1.0*, a FRET-based K^+^ indicator targeted to the mitochondrial matrix. At time points indicated in the panel, either ATP, or gramicidin, an ionophoric peptide for K^+^, were administered to the cells. The dashed line indicates the onset of the K^+^ decrease under gramicidin. Data represents average ± SEM, n = 9 independent experiments per genotype. Scalebar = 20 µm. (**E**) Corresponding basal FRET-ratio values (timepoints 0 – 5) of curves shown in (D). Bars represent average ± SEM, n = 9 ***p≤0.001, unpaired t-test. (**F**) Representative FRET (left panel, yellow, left), CFP (left panel, cyan, middle) and pseudocoloured FRET-ratio images (left panel, 16-colors, right) and FRET-ratio signals over-time (right panel) of MMTV-PyMT WT (black) and MMTV-PyMT IK KO cells (red) expressing *cyto lc-LysM GEPII 1.0*, a FRET-based K^+^ indicator targeted to the cytosol. At time points indicated in the panel gramicidin was administered to the cells. The dashed line indicates the onset of the K^+^ decrease under gramicidin. Data represents average ± SEM, n = 6 independent experiments per genotype. Scalebar = 20 µm. (**G**) Demonstrates corresponding basal FRET-ratio values (timepoints 0 – 5) of curves shown in (F). Bars represent average ± SEM, n = 6 ***p≤0.001, unpaired t-test.


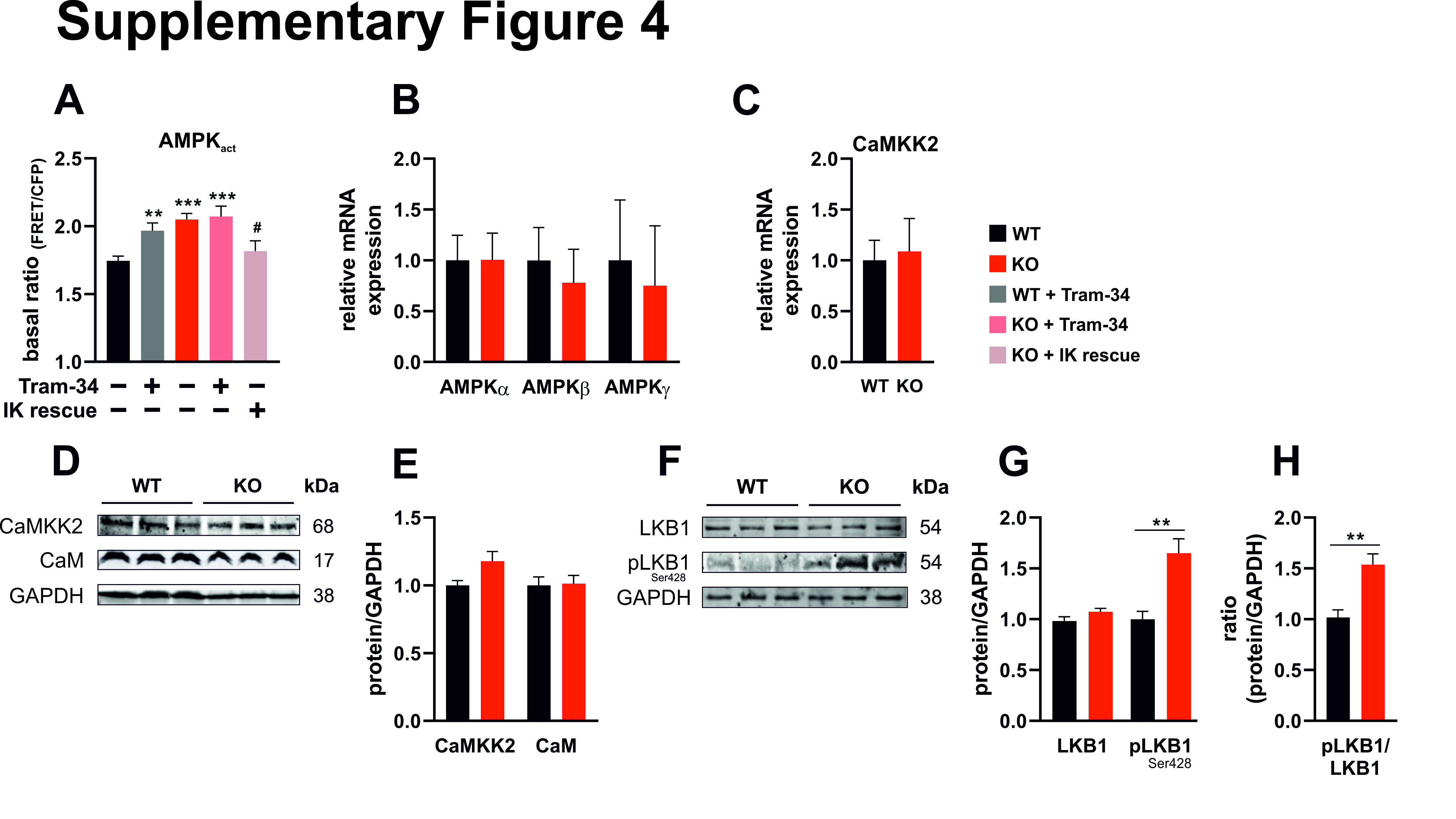


**Figure S4.** *Investigation of upstream AMPK pathways.* (**A**) Basal FRET-ratio signals of MMTV-PyMT WT (black and grey bar) and MMTV-PyMT IK KO cells (red, salmon and bright red bar) expressing *AMPKAR*, a FRET-based AMPK activity reporter (AMPK_act_). Cells were either treated with DMSO, TRAM-34 (for 48 h) or transiently transfected with IK as indicated (IK rescue). Bars represent average ± SEM, n = 5 **p≤0.01, ***p≤0.001, ^#^p≤0.05 (^#^ = compared to untreated condition of same genotype), one-way ANOVA. (**B**) qRT-PCR analysis of AMPK subunit genes (α-γ) obtained from cDNA of MMTV-PyMT WT (black bar) and MMTV-PyMT IK KO (red bar). Bars represent average ± SEM, n = 7 (WT), n = 6 (IK KO), unpaired t-test. (**C**) qRT-PCR analysis of CaMMK2 from cDNA of MMTV-PyMT WT (black bar) and MMTV-PyMT IK KO (red bar). Bars represent average ± SEM, n = 8, unpaired t-test. (**D**) Western blot analysis of CaMMK2, CaM and GAPDH of protein lysates obtained from MMTV-PyMT WT (left) and MMTV-PyMT IK KO (right) cells. Data represents average ± SEM of n = 6 independent experiments per genotype. (**E**) Quantification of Western blot band intensities as shown in (D) and normalization on GAPDH of MMTV-PyMT WT (black bar) and MMTV-PyMT IK KO (red bar). Bars represent average ± SEM, n = 6, unpaired t-test. (**F**) Western blot analysis of LKB1, phosphorylated LKB1 at Ser 428 and GAPDH of protein lysates obtained from MMTV-PyMT WT (left) and MMTV-PyMT IK KO (right) cells. Data represents average ± SEM of n = 8 independent experiments per genotype. (**G**) Quantification of Western blot band intensities as shown in (F) and normalization on GAPDH of MMTV-PyMT WT (black bar) and MMTV-PyMT IK KO (red bar). Bars represent average ± SEM, n = 8 **p≤0.01, unpaired t-test. (**H**) Demonstrates the ratio of phosphorylated LKB1 to whole LKB1 intensities normalised on GAPDH of MMTV-PyMT WT (black bar) and MMTV-PyMT IK KO (red bar). Bars represent average ± SEM, n = 8 **p≤0.01, unpaired t-test.


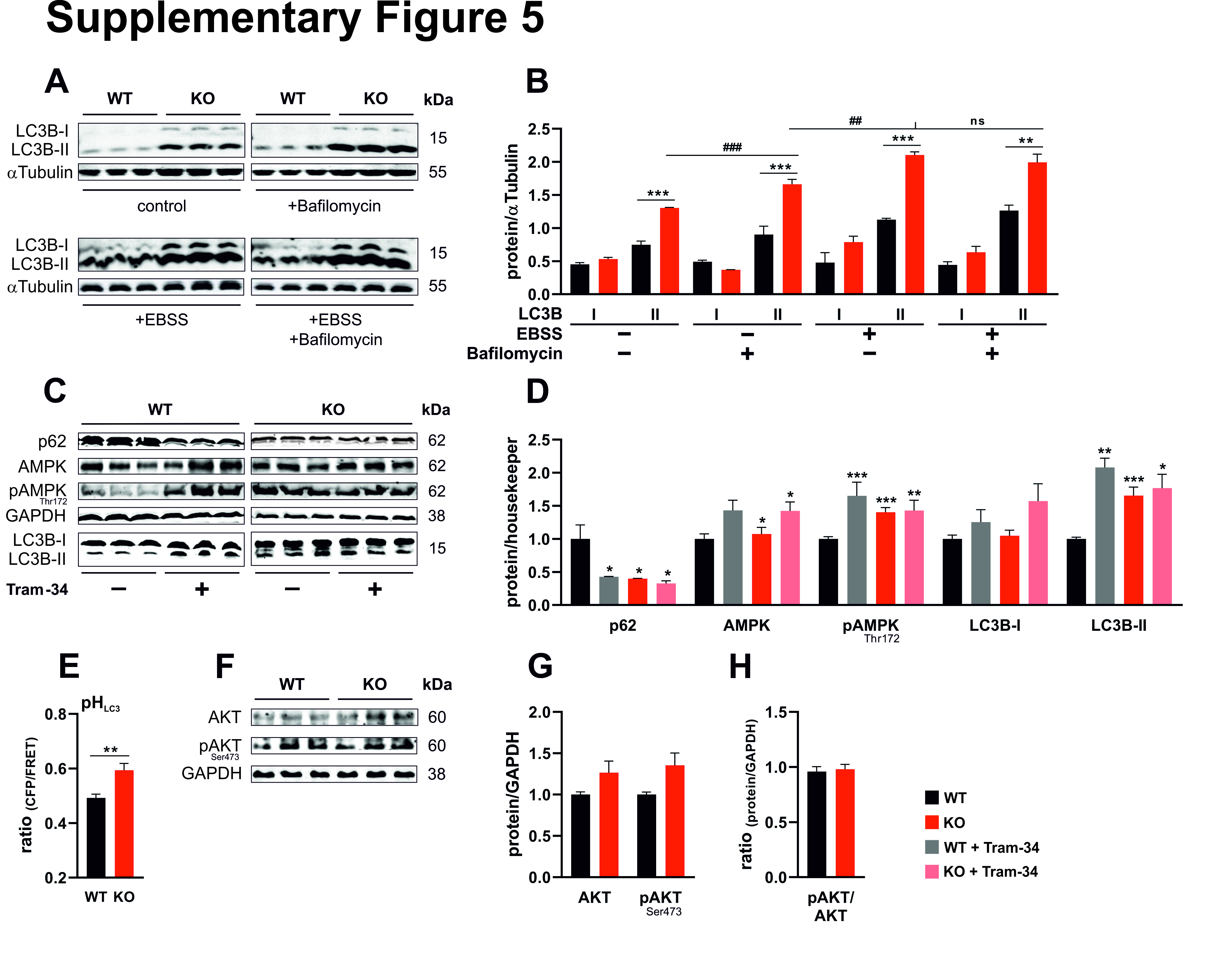
**Figure S5.** *Genetical- and pharmacological IK inhibition promote autophagic flux.* (**A**) Western blot analysis of αTubulin and LC3B (I + II) of protein lysates obtained from MMTV-PyMT WT (left) and MMTV-PyMT IK KO (right) cells either treated with the V-ATPase inhibitor Bafilomycin, the starvation medium EBSS or a combination of both. Data represents average ± SEM of n = 6 independent experiments per genotype. (**B**) Quantification of Western blot band intensities as shown in (A) and normalization of LC3B (I + II) on αTubulin of MMTV-PyMT WT (black bar) and MMTV-PyMT IK KO (red bar). Bars represent average ± SEM, n = 6 **p≤0.01, ***p≤0.001, ^##^p≤0.01, ^###^p≤0.001 (^#^ = compared to untreated condition of same genotype), 2-way ANOVA. (**C**) Western blot analysis of P62, AMPK, phosphorylated AMPK at Thr 172, GAPDH and LC3B (I + II) of protein lysates obtained from MMTV-PyMT WT (black bar) and MMTV-PyMT IK KO (red bar) either treated with DMSO or 2 µM Tram-34 (for 48 h) as indicated. Data represents average ± SEM of n = 6 independent experiments. (**D**) Quantification of Western blot band intensities as shown in (D) and normalization on GAPDH of MMTV-PyMT WT (black and grey bars) or MMTV-PyMT IK KO cells (red and salmon bars). Cells were either treated with DMSO as a vehicle control (black and red bars) or 2 µM TRAM-34 (for 48 h, grey and salmon bars). Bars represent average ± SEM, n = 6 *p≤0.05, **p≤0.01, ***p≤0.001, unpaired t-test. (**E**) Basal FRET-ratio signals (measurement over 5 min) of MMTV-PyMT WT (black bar) and MMTV-PyMT IK KO cells (red bar) expressing *pH-Lemon LC3B*, a FRET-based pH indicator fused to LC3B for the visualization of autophagy. Bars represent average ± SEM, n = 3 **p≤0.01, unpaired t-test. (**F**) Western blot analysis of AKT, phosphorylated AKT at Ser 473 and GAPDH of protein lysates obtained from MMTV-PyMT WT (left) and MMTV-PyMT IK KO (right) cells. Data represents average ± SEM of n = 9 independent experiments per genotype. (**G**) Quantification of Western blot band intensities as demonstrated in (G) and normalization on GAPDH of MMTV-PyMT WT (black bar) and MMTV-PyMT IK KO (red bar). Bars represent average ± SEM, n = 9, unpaired t-test. (**H**) Ratio of phosphorylated AKT to whole AKT intensities normalised on GAPDH of MMTV-PyMT WT (black bar) and MMTV-PyMT IK KO (red bar). Bars represent average ± SEM, n = 9, unpaired t-test.

**Full and uncropped Western blot for Figure 4 A**

Red boxes display used WB bands on figure

AMPK (62 kDa) and GAPDH (38 kDa)


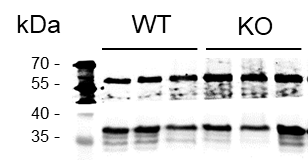


AMPK

pAMPK (Thr172, 62 kDa) and GAPDH (38 kDa)


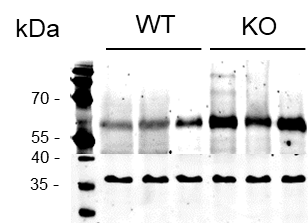


GAPDH

pAMPK

**Full and uncropped Western blot for Figure 5 A**

Red boxes display used WB bands on figure

LC3B (14, 17 kDa) and αTubulin (55 kDa)


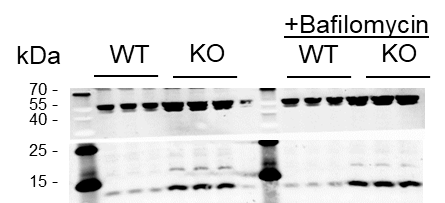


LC3B-I

LC3B-II

αTubulin


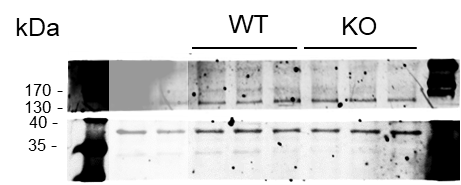
ULK1 (140 kDa) and GAPDH (38 kDa)

ULK1

pULK1 (Ser555, 140 kDa) and GAPDH (38 kDa)


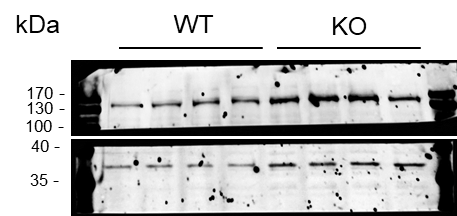


pULK1

p62 (62 kDa) and GAPDH (38 kDa)


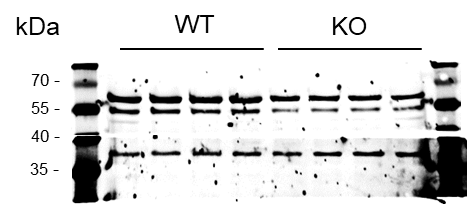


GAPDH

p62

**Full and uncropped Western blot for Figure S4 D**

Red boxes display used WB bands on figure

CaM (17 kDa) and GAPDH (38 kDa)


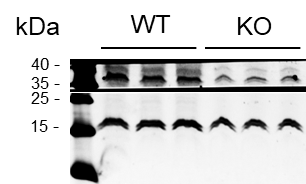


CaM


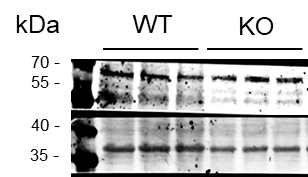
CaMMK2 (68 kDa) and GAPDH (38 kDa)

CaMKK2

GAPDH

**Full and uncropped Western blot for Figure S4 F**

Red boxes display used WB bands on figure

LKB1 (54 kDa) and GAPDH (38 kDa)


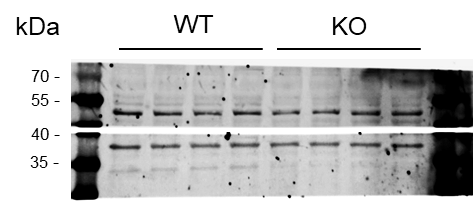


LKB1

GAPDH


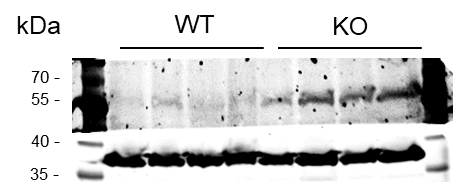
pLKB1 (Ser428, 54 kDa) and GAPDH (38 kDa)

pLKB1

**Full and uncropped Western blot for Figure S5 A**

Red boxes display used WB bands on figure

LC3B (14, 17 kDa) and αTubulin (55 kDa)


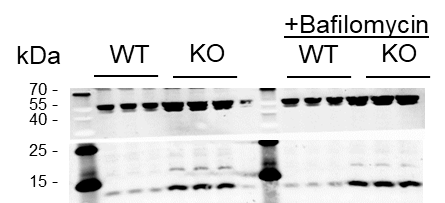


LC3B-I

LC3B-II

αTubulin


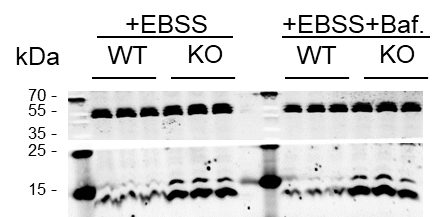


LC3B-I

LC3B-II

αTubulin

**Full and uncropped Western blot for Figure S5 C**

Red boxes display used WB bands on figure

P62 (62 kDa) and GAPDH (38 kDa)


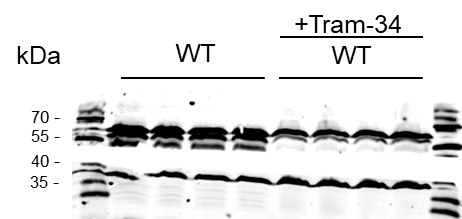

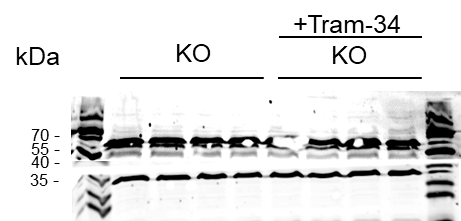


GAPDH

p62

AMPK (62 kDa) and GAPDH (38 kDa)


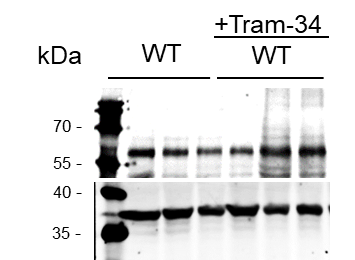

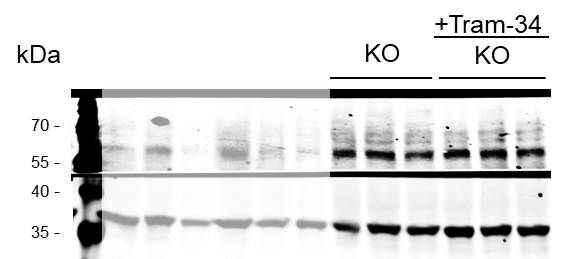


AMPK

pAMPK (Thr172, 62 kDa) and GAPDH (38 kDa)


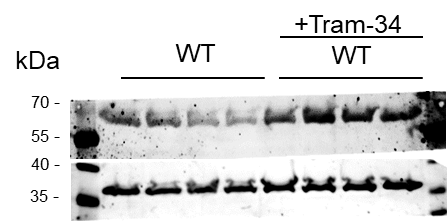

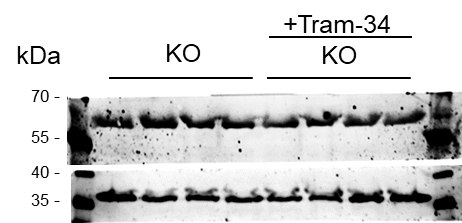


pAMPK

LC3B (14, 17 kDa) and αTubulin (55 kDa)


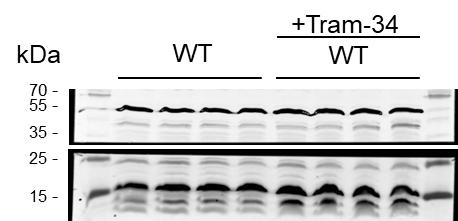

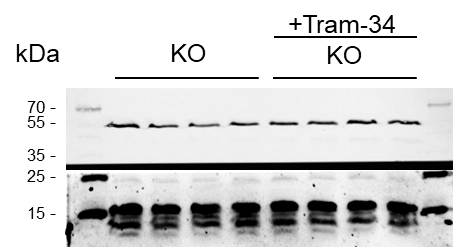


LC3B-I

LC3B-II

**Full and uncropped Western blot for Figure S5 F**

Red boxes display used WB bands on figure

AKT (60 kDa) and GAPDH (38 kDa)


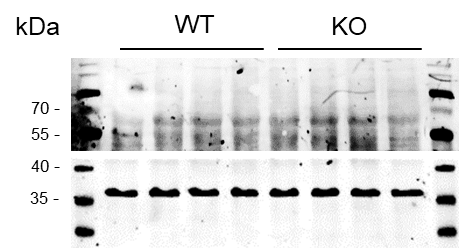


AKT

GAPDH

pAKT (Ser473, 60 kDa) and GAPDH (38 kDa)


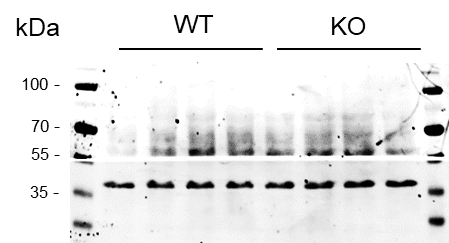


pAKT
